# Colorectal Cancer and Immunity: From the Wet Lab to Individuals

**DOI:** 10.3390/cancers13071713

**Published:** 2021-04-04

**Authors:** Elodie Pramil, Clémentine Dillard, Alexandre E. Escargueil

**Affiliations:** 1 Sorbonne Université, INSERM U938, Centre de Recherche Saint-Antoine, F-75012 Paris, France; elodie.pramil@hotmail.fr (E.P.); clementine.dillard@inserm.fr (C.D.); 2 Alliance Pour la Recherche en Cancérologie—APREC, Tenon Hospital, F-75012 Paris, France

**Keywords:** colorectal cancer, immunotherapy, methods

## Abstract

**Simple Summary:**

Tackling the current dilemma of colorectal cancer resistance to immunotherapy is puzzling and requires novel therapeutic strategies to emerge. However, characterizing the intricate interactions between cancer and immune cells remains difficult because of the complexity and heterogeneity of both compartments. Developing rationales is intellectually feasible but testing them can be experimentally challenging and requires the development of innovative procedures and protocols. In this review, we delineated useful in vitro and in vivo models used for research in the field of immunotherapy that are or could be applied to colorectal cancer management and lead to major breakthroughs in the coming years.

**Abstract:**

Immunotherapy is a very promising field of research and application for treating cancers, in particular for those that are resistant to chemotherapeutics. Immunotherapy aims at enhancing immune cell activation to increase tumor cells recognition and killing. However, some specific cancer types, such as colorectal cancer (CRC), are less responsive than others to the current immunotherapies. Intrinsic resistance can be mediated by the development of an immuno-suppressive environment in CRC. The mutational status of cancer cells also plays a role in this process. CRC can indeed be distinguished in two main subtypes. Microsatellite instable (MSI) tumors show a hyper-mutable phenotype caused by the deficiency of the DNA mismatch repair machinery (MMR) while microsatellite stable (MSS) tumors show a comparatively more “stable” mutational phenotype. Several studies demonstrated that MSI CRC generally display good prognoses for patients and immunotherapy is considered as a therapeutic option for this type of tumors. On the contrary, MSS metastatic CRC usually presents a worse prognosis and is not responsive to immunotherapy. According to this, developing new and innovative models for studying CRC response towards immune targeted therapies has become essential in the last years. Herein, we review the in vitro and in vivo models used for research in the field of immunotherapy applied to colorectal cancer.

## 1. Introduction

Colorectal cancer (CRC) is the third most commonly diagnosed malignancy worldwide, and the second leading cause of cancer related-deaths among men and women with 1.8 million estimated cases and more than 800,000 deaths annually [1]. As the disease mostly progresses indolently at the initial stages, becomes symptomatic late, and is often diagnosed at an advanced stage (about 35% of patients presenting with a metastatic cancer). This issue is of importance because the prognosis for CRC patients is strongly dependent on the stage of the tumor at diagnosis [2]. Following the Tumor Node Metastasis (TNM) staging, the 5-year survival rates following surgical removal of tumors for localized (stage I), regional (stages II and III) and metastatic (stage IV) diseases reach in the USA 90, 72, and 14%, respectively [3]. For stage I and most stage II CRCs, the standard of care is surgery alone [2]. For high-risk stage II and stage III CRCs, surgical removal is followed by adjuvant 5-fluoruracil (5-FU) or capecitabine-based chemotherapy [4,5]. For metastatic disease, surgical removal of the primary and/or distant lesions is followed by therapies using a set of chemotherapies and targeted agents [2]. However, as mentioned before, the prognosis of patients with metastatic CRC (mCRC) remains poor, with a median overall survival (OS) of about 30 months [6]. In addition to the TNM classification, recent advances have led to the development of immune-based classifications of colorectal tumors. Indeed, numerous reports demonstrated that an enhanced T-lymphocytic infiltration in tumor tissues is associated with an improved prognosis [7,8]. However, the composition of the tumor microenvironment (TME) varies substantially between colorectal tumors [2,9]. Thus, in an effort to translate these findings to the clinic, an international consortium developed the “Immunoscore” [10]. This scoring system is based on the histological quantification and localization of cytotoxic and memory T-cells in the center of the tumor and invasive margin. Importantly, time to recurrence was significantly improved in patients with stage I–III colon cancer presenting a high “Immunoscore” [11]. These observations thus supported the role of this scoring system in providing a reliable estimate of the risk of recurrence in patients with colon cancer and its additional prognostic value when combined with conventional TNM-staging [9]. They also underline the impact of immune cell infiltration on CRC outcome, thus opening new therapeutic opportunities.

In addition to the characterization of immune cells infiltrates in CRC, significant research has also helped in the last years to better understand the complex interplay between cancer and immune cells. This knowledge has led to the emergence of novel immunotherapies including the development of immune checkpoint inhibitors (anti-CTLA-4, anti-PD-1, and anti-PD-L1 monoclonal antibodies). These molecules have dramatically changed the therapeutic situation for several types of cancer [12]. However, for mCRC, only few objective responses have been observed in unselected colorectal cancer patients. Long-lasting responses were only restricted to 4 to 5% of patients who presented tumors harboring microsatellite instability (MSI-H) and/or mismatch repair deficiency (dMMR) [12,13,14,15]. For this small subset of patients, the therapeutic scenario was nonetheless significantly changed thanks to the introduction of immune checkpoint inhibitors. In 2020, pembrolizumab (anti PD-1) was approved by the U.S. Food and Drug Administration (FDA) for the first-line treatment of patients with unresectable or metastatic dMMR CRC. The success of immunotherapies for treating metastatic dMMR CRC also paved the way for clinical research aiming at introducing immunotherapy in the adjuvant setting used for treating patients with localized MSI/dMMR CRC [14].

This success for this specific subtype of CRC is, however, not surprising in terms of biological understanding. Indeed, MSI tumors are known for being highly intruded by tumor-infiltrating lymphocytes (TILs) such as CD8+ cytotoxic lymphocytes, Th1-activated cells that produce IFNγ, and CD45 RO+ T memory cells [8,16,17]. This phenomenon is explained by the hypermutated phenotype of these tumors, leading to high mutational burden (TMB) with highly immunogenic neoantigens as a consequence of a large number of deletions, insertions, and frameshift mutations accumulated during cancer cell replication [12,14,15]. The accumulation of tumor-associated neoantigens indeed favors the identification of cancer cells by the host immune system [18,19]. This hypothesis was recently confirmed in a controlled murine syngeneic model of CRC [20]. By genetically inactivating DNA mismatch repair in an otherwise MMR proficient (pMMR) cell line, the authors clearly demonstrated that MMR loss caused a tumor hyper-mutated status associated with an increased load of tumor neoantigens. In turn, those triggered long-lasting immune surveillance that could be further enhanced by immune modulators [20]. Importantly, MSI/dMMR tumors are often associated with an upregulation of checkpoint inhibitors that exhaust intra-tumor cytotoxic T lymphocytes and consequently protect MSI/dMMR cancer cells from their hostile immune microenvironment [21,22]. Together, these made metastatic MSI/dMMR tumors a valuable candidate for immune checkpoint inhibitors (ICI).

Unfortunately, this type of ICI responsive tumors (e.g. MSI/dMMR) represents only 15 to 20% of total CRC and about 4% of stage IV CRC [12,23,24]. Therefore, a vast majority of mCRC is cold refractory to this therapeutic strategy. As with most cancers, CRC is a genetically heterogeneous disease. However, heterogeneity also emerges from the composition of the surrounding tissue and cells, commonly called the tumor microenvironment. This includes epithelial cells, blood and lymphatic vessels, stromal and infiltrating immune cells, as well as extracellular components (e.g., chemokines, cytokines, and extracellular matrix) [9]. This general context and the subsequent crosstalks established between TME and tumor cells are key features to determine the effect of infiltrating immune cells on clinical outcome. According to these observations, and based on both tumor and infiltrating stroma gene expression profiles, a consensus molecular subtype (CMS) classification has been set up in the last years [25]. According to it, four major groups were distinguished: CMS1 (approximately 14% of cases) are hypermutated tumors, mostly with MSI-H features and showing a robust immune cells infiltration; CMS2 (approximately 37% of cases) are canonical CRC tumors characterized by the activation of the Wnt and Myc pathways; CMS3 (approximately 13% of the cases) are tumors frequently mutated in *KRAS* and displaying a deregulated cancer cell metabolism; CMS4 (approximately 23% of the cases) are mesenchymal tumors characterized by transforming growth factor beta (TGF-β) pathway activation, enhanced angiogenesis, stromal activation, and inflammatory infiltrate [9,12,25]. The 13% of missing samples corresponded to tumors with mixed features (13%) [25]. Interestingly, in this classification, CMS1 and CMS4 were considered as “hot” tumors with an intense immune infiltration, whereas CMS2 and CMS3 were defined as “cold” tumors with a lack of immune activation [12]. However, CMS4 tumors, despite their immune infiltration, displayed the worse overall and relapse-free survival [25]. This is explained by the specific immune infiltrate seen in these tumors mostly composed of T regulatory cells (Treg), myeloid-derived suppressor cells (MDSCs), and monocyte-derived cells. This inflamed immune-tolerant TME is characterized by marked upregulation of immunosuppressive factors, such as TGF-β, Vascular endothelial growth factor (VEGF), and CXCL12 [26]. On the contrary, CMS1 CRC has a diffuse immune infiltrate with notable CD8+ TILs. As discussed above, those MSI/dMMR tumors also upregulate immune checkpoint molecules (CTLA-4, PD-1, PD-L1) [21,22,26]. Though the use of ICIs can activate an effective antitumor immune response for CMS1 but not for CMS4 CRC subtypes [12], nonetheless, strategies aiming at targeting the TGF-β and/or VEGF pathways might prove useful for CMS4 CRC [27,28]. In contrast to CMS1 and CMS4 tumors, CMS2 and CMS3 tumors were defined as “immune desert” cancers [12]. Different mechanisms can be responsible for this phenomenon, including lack of major histocompatibility complex (MHC) class I molecules and/or upregulation of nonclassical human leukocyte antigens (HLA) [9,12]. Interestingly, targetable oncogenic-driven cancer cell pathways have been identified as potential sources of the above immune evasion processes. For example, MEK inhibition has been shown to rescue low class I MHC expression and augment anti-tumor T-cell immunity [29]. However, acting on the sole tumor compartment is likely not to be sufficient to overcome resistance in “immune desert” tumors. To that end, combinatorial strategies aiming at increasing immune cells infiltrate and/or neoantigens generation or release have been proposed to synergize with immunotherapies [15]. For example, current chemotherapies, like oxaliplatin and 5-FU can improve TME immune-competency by inducing immunogenic cell death and/or depleting MDSCs [30,31,32]. VEGF-targeted therapy can also improve TME immune competency by reducing the proportion and number of Tregs in CRC murine tumors as well as in the peripheral blood of patients with mCRCs [33]. On the other hand, cetuximab which targets the extracellular domain of epidermal growth factor receptor (EGFR) might promote activation of the immune response in CRC patients in addition to its direct action on cancer cells [34]. In the last years, several clinical trials have been launched to evaluate strategies combining chemotherapies and targeted therapies to extend the efficacy of immunotherapy to pMMR CRC [15]. However, to date, those approaches and rationales have not been successfully transformed in terms of clinical benefits [15]. Further insights into the molecular mechanisms underlying the immune competence and/or immunotherapy resistance are therefore urgently needed for developing predictive biomarkers and/or improving pharmacological combination strategies for mCRC resistant to immunotherapy. To this end, substantial help is awaited from translational research with the aim of turning all cold CRC into hot responsive immunogenic tumors.

Herein, we review the in vitro and in vivo models used for research in the field of immunotherapy applied to CRC. We discuss their useful meaning and propose to define the most accurate approaches to expand our knowledge on immunological-based therapies in pMMR colorectal cancer with a special emphasis on models allowing a better characterization of the resistance mechanisms, as well as the identification of predictive biomarkers and the assessment of novel combinatorial therapeutic strategies.

## 2. In Vitro Models for Immunotherapy Studies

### 2.1. 2-Dimensional Methods

Tumors, including CRC, are not composed of homogeneous cell populations but contain a multitude of cells with different characteristics [35]. This heterogeneity leads to different treatment responses within the tumor itself as well as between patients and has to be considered early while developing new therapies. Cancer cell lines are a widely used tool for pre-clinical in vitro research. Their major advantage is their simple manipulation. Due to the heterogeneity of CRC, there is a multitude of derived cell lines available to date with different molecular patterns. Hence, the selection of the most relevant model is a crucial step during the development of new pharmaceutics. Numerous multi-omics studies have dealt with the analysis and classification of CRC. In particular, following the CMS classification [25], Berg et al. analyzed 34 CRC cell lines and classified them among the 4 CMS groups mentioned above, bringing new resources for CRC model selection (Table 1) [36]. As discussed, MSI/dMMR tumors are preferentially immunogenic, heavily infiltrated by lymphocytes and good responders to immunotherapies [37]. Accordingly, in vitro studies on CMS1 CRC cell lines gave interesting responses to immunotherapies. However, to better understand the heterogeneous behaviors seen in tumors, experiments need to be extended to other cell lines. More specifically, cells corresponding to the major types of mCRC should carefully be characterized to respond to the huge therapeutic challenge we are facing now [38]. The initial choice of the cell line is therefore an important criterion. Testing and comparing responses in cells originating from different CMS clusters will therefore prove useful for assessing the underlying molecular mechanisms of resistance to immunotherapies. We thereafter discuss different experimental models based on the use of commercially available cancer cell lines for the study of immunotherapies in vitro.

#### 2.1.1. Cancer Cells: Secretome Assessment

**Figure 1:**
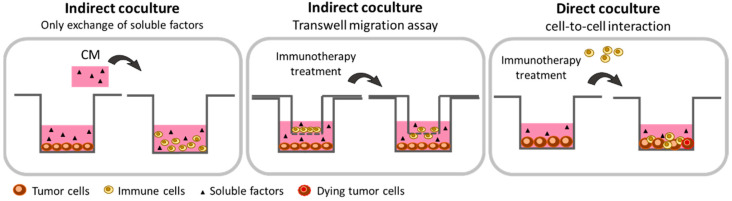
Schematic representation of the 2-dimensional (2D) co-culture methods that are suitable for in vitro study of immunotherapies. The in vitro 2D co-cultures using commercialized cell lines are a first approach to evaluate the activation, migration, or cytotoxic potential of immune cells following an immunomodulatory treatment because of its simplicity to set up, its low cost, and its reproducibility. Indirect co-culture consists of the transfer of conditioned medium from one cell to another. This allows the effects of soluble factors on immune cells biology to be studied. The indirect co-culture method using the Transwell assay allows the study of the migratory capacity of immune cells in the presence of tumor-derived conditioned media. Finally, the direct co-culture assay permits cell-to-cell interactions, thus allowing studies on immune cells activation and cytotoxic activity towards tumor cells.

During culturing, cells naturally release proteins, soluble factors, exosomes, or microvesicles capable to act on cell interaction, proliferation, death, metabolism, or drug resistance [39]. In the context of immunotherapy, transferring a conditioned medium (CM) from one cell culture to another is a simple experiment to address the effect of the cancer cells’ secretome on immune cells’ phenotype (Figure 1) [40]. In the recent years, numerous studies have focused on the establishment of therapeutic strategies for converting tumor-associated macrophages (TAMs) displaying an immunosuppressive M2 phenotype into a pro-inflammatory M1 phenotype [41,42,43]. In order to generate TAMs in vitro, the use of tumor-based CM has proven to be an effective approach. Several research groups have indeed demonstrated that TAMs, differentiated in tumor-based CM, display the same genetic, phenotypic, and functional characteristics as the tumor-associated macrophages derived from patients [44,45]. Benner et al. used conditioned media derived from two breast cancer cell lines which was complemented with a cocktail of cytokines (IL-4, IL-10, M-CSF) and incubated it with healthy donor monocytes to successfully generate tumor-associated macrophages (TAMs). Those TAMs which differentiated in M2 macrophages showed an increased co-expression of the CD163/CD206 TAM surface markers as well as several functional TAM markers. Importantly, those TAMs also secreted factors in vitro able to promote tumor cells survival and growth [46]. In a similar approach using tumor-based CM, Dong et al. also produced TAMs and demonstrated that the immunocomplex formed between lactoferrin and anti-lactoferrin was capable of converting M2-TAMs towards the M1 phenotype [47]. These studies demonstrate the potential of producing in vitro TAMs for studying new immunological opportunities. For CRC, this strategy also was successful since CRC-based CM prepared from 4 distinctive cell lines was able to activate and induce differentiation of the human monocytic cell line THP-I towards a TAM-associated phenotype displaying immunosuppressive properties [48]. 

In addition to TAMs, T lymphocytes are another immune cell population that is highly targeted by immunotherapies. Adil et al. studied the effects of cancer cell based-CM on Peripheral Blood Mononuclear Cell (PBMCs) originating from healthy donors. They showed an anti-proliferative effect of both MCF7 and HeLa conditioned media. However, CM prepared from the leukemic K562 cell line demonstrated a pro-proliferative effect on PBMC associated with an increased expression of Treg markers and of the CD4+/Helios+ subpopulation. These results correlate with the induction of immunosuppressive functions of PBMC promoted by CM [49]. Similarly, it was shown that CM prepared from the RENCA mouse kidney cancer cell line converted CD4+CD25- T lymphocytes into CD4+CD25+ Treg cells [50]. Together, these methodological approaches underline the important crosstalk existing between immune and tumor cells and the influence that secreted soluble factors can exert on the fate of immune cells. This, combined with the development of new omics technologies, can help future studies aiming at identifying new targetable immunologic molecules involved in cell–cell trans-communication. Such approaches have yet been useful for CRC by demonstrating the capacity of the Treg supernatant to enhance chemoresistance [51]. Recently, conditioned media were prepared from rectal cancer and non-cancer control biopsies and 19 oversecreted inflammatory proteins were identified in the rectal cancer secretome [52]. By comparing CRC-CM-induced effects on immune cells, and conversely by comparing immune cell-CM-induced effects on colorectal cancer cells, new interventional opportunities can therefore be identified and secretome components targeted to eventually be blocked [53]. Alternatively, targetable cellular pathways involved in the secretion of specific soluble factors having an immunogenic potential might directly be identified and pharmacologically evaluated through new screening approaches in CRC tumor cells selected among those classified as CMS2-4.

#### 2.1.2. Co-Culture with Paracrine Interaction: The Transwell Technology

Immunotherapies are ineffective for most pMMR CRC cancer because of the poor number of infiltrating immune cells in the tumor microenvironment [54]. The development of immunotherapies able to promote migration and recruitment of immune cells within the tumor microenvironment is thus essential [8,55,56]. The Transwell technology can be used in vitro to study the impact of immunotherapeutic molecules to act on the ability of cancer cells to attract immune cells. The Transwell consists of an upper insert containing a permeable membrane allowing the exchange of soluble factors and/or cell migration (Figure 1). Several Transwell pore sizes are indeed commercially available. Membrane with a pore size of 0.4 µm exclusively permits measuring the exchange of soluble factors like cytokines between the two compartments. In contrast, a larger pore size allows cells to migrate through the membrane. For the study of human-derived immune cell migration, a 3 to 5 µm pore size membrane is usually sufficient [57,58,59]. In Transwell coculture, immune cells (PBMCs or isolated subpopulations of immune cells) are seeded onto the upper layer of the insert, while the tested molecules, CM, or attached cells are deposited in the lower chamber. Tumor cells can then be stimulated or treated (chemotherapeutics or radiotherapy) before positioning the Transwell upper layer to trigger the secretion of soluble factors. After diffusion throughout the well, those factors can reach immune cells with their subsequent activation, proliferation, cytokines production, and/or migration easily monitored (Figure 1). Hence, activation can be studied by flow cytometry through the expression of specific surface markers. Proliferation can be assessed by counting cells in the upper chamber. Cytokine secretion can be quantified by ELISA assay and/or cytokine array [60]. Finally, immune cells migration can be evaluated either after fixation, coloration, and counting with a microscope [61,62] or by flow cytometry, after Transwell centrifugation and cell harvesting, with antibodies directed against specific surface antigens. The percentage of each cell subpopulation can thus be precisely determined [63,64]. Transwell is commonly used to test chemotaxis of immune or cancer cells. For example, Harlin et al. demonstrated that chemokines (CCL2, CCL3, CCL4, CCL5, CXCL9, and CXCL10) are able to induce migration of CD8+ T-cells from the upper to the lower chamber of the Transwell. They also demonstrated that, in contrast to the culture medium alone, the presence of M537-CM melanoma cells in the lower compartment stimulates the recruitment of CD8+ T-cells [63]. Similarly, Hennel et al. used the Transwell technology to study the ability of breast cancer cells dying after radiotherapy to release factors capable to stimulate monocyte migration. To do so, they seeded in the upper insert THP-1 macrophages while supernatants from mock-irradiated and irradiated breast cancer cells were put in the lower chamber. This approach allowed them to demonstrate that radiation-induced necrosis of HCC1937 cells is particularly efficient for stimulating THP-1 cell migration and identifying apyrase-sensitive nucleotides as molecules responsible for attracting monocytes [65]. Transwell assays were also used to study the migratory capacity of mast cells in CRC [66]. This immune cell type is one of the earliest to be recruited during CRC tumorigenesis. In this work, the authors plated human CD34+-derived mast cells in the upper chamber of the Transwell while CM prepared from either HT29 or Caco2 CRC cells were positioned in the lower chamber. Their results demonstrated a significant increase of mast cell migration in both conditions. However, mast cells’ chemo-attraction originated from two distinct mechanisms according to the CRC cell line used. The stem cell factor (SCF) seems to be involved in the Caco-2-CM while CCL15 chemokine is responsible for the mast cell migration in HT29-CM [66]. These works highlight the effectiveness of using the Transwell assay to deal with the migratory functions of immune cells and could be an asset for the understanding of the migration mechanisms following treatment by immunotherapies.

#### 2.1.3. Co-Culture with Direct Cell-to-Cell Interaction

The use of direct co-culturing conditions allows physical contact between tumor and immune cells. This approach should be considered as an alternative method for studying in vitro anti-cancer immunotherapies [67]. It indeed allows the role of cell–cell physical contact in addition to the action of secreted soluble factors to be evaluated. It also the direct cytotoxic activities of immune cells towards cancer cells to be evaluated (Figure 1). Co-cultures can be performed with either PBMC from healthy donors, mice, patients’ peripheral blood, or immune cells collected and isolated from colorectal carcinoma specimens. Prior to establish the co-cultures, immune cells can be sorted out in order to isolate specific immune cell subpopulations (LT, LB, NK, DC, Mono) or differentiated in vitro (macrophages) [68,69]. However, an important issue is the need to precisely establish the ratio that is used in the co-culture experiment between tumor cells and immune cells. Moreover, defining the precise cytokines/antibodies cocktail required for activating immune cells can be particularly tricky. Anti-CD3/CD28 beads, IL-12, and M-CSF or GM-CSF are usually used for activating T-cells, NK-cells, and macrophages, respectively [70,71,72]. In addition, defining the incubation time of the co-culture prior to performing analyses is an important issue and is likely to depend on the tumor cell type used. In that sense, a detailed protocol used for performing co-cultures of tumor cells with T-cells was described by Melief et al. [73]. In addition, Minute et al. performed co-cultures combining modified MC38 CRC murine cells (MC38EGFROVA) with either cytotoxic T lymphocytes (CTL) or activated NK-cells. Interestingly, prior to establishing co-cultures, tumor cells were here pre-treated with IFN-γ in order to increase their expression of MHC-class I. Human gp100 peptide was also added to load MHC-class I. The first co-culture was then established with CD8+ splenocytes which were preactivated in vivo in mice. Activated splenocytes and preconditioned MC38 were then co-cultured for 3 days at a 10:1 ratio, in the presence of IL-2 and human gp100. The second co-culture model was established with NK-cells which were also preactivated in vivo in mice. In that case, NK-cells were co-cultured for 3 days with MC38 cells which were not preconditioned, at a 5:1 ratio and in presence of IL-2. At the end of the co-cultures, the authors highlighted the presence of two alarmins in the extracellular compartment, HMGB1, which was released in the culture medium and the calreticulin which was exposed on the cell surface. This study presented evidence that T- and NK-cells induce features of immunogenic cell death (ICD) on tumor cells and that in vitro co-culture can trigger immune response against tumor cells. Importantly, in the same study, the authors demonstrated the same capacity of CTL and NK-cells to induce ICD on the human CRC cell line HT29. In the presence of a bispecific antibody EpCAM-CD3ε, HMGB1 and calreticulin were indeed exposed on the HT29 cells when co-cultured with CTL or NK-cells (preactivated by IL-2 and IL-15). However, the ratio used here (1:1) was strikingly different from those used for the murine cells [74]. This underlines the difficulties of setting up general protocols and the sometimes difficult interpretation in terms of biological relevance. In a similar context, others performed co-cultures of HT29 CRC cells with CD8+ T-cells isolated from either healthy donors, CRC patients’ peripheral blood, or from tumor immune infiltrates. The CD8+ T-cells were then stimulated for 2 h in the presence of anti-CD3/CD28 beads and then directly and indirectly (Transwell) co-cultured with HT29 cells (4:1 ratio) for 48 h in the presence of anti-CD3/CD28 beads. In this study, the authors evaluated the effect of the indirect pharmacological inhibition of Notch by the γ-secretase inhibitor DAPT on the anti-tumor immunity. Interestingly, they evidenced an increased production of pro-inflammatory cytokines (IFN-γ, IL-1β, IL-6, TNF-α) in the supernatant of both direct and indirect co-cultures when HT29 cells were co-cultured with either peripheral or infiltrated tumor CD8+ T-cells in presence of DAPT. However, a significant increase in CD8+ T-cell-induced HT29 cell death was only observed when co-cultures were made with CD8+ T-cells which were purified from colorectal carcinoma, not CD8+ T-cells isolated from peripheral blood. This phenomenon was also reported only when cells were co-cultured in conditions permitting direct contacts between cells. Remarkably, in the absence of Notch inhibition, this study also demonstrated a greater cytotoxic effect on HT29 cells of peripheral CD8+ T-cells isolated from healthy donors compared to peripheral CD8+ T-cells isolated from CRC patients. Thus, this study evidenced the immunosuppressive potential of Notch signaling in CRC and demonstrated the in vitro ability of Notch inhibition to stimulate and restore anti-tumor immunity [75]. Co-cultures with direct cell-to-cell interaction were also used for deciphering the mechanism of action of the TIGIT immune checkpoint (present on T-cells’ surface) and its role in the impairment of metabolism and function of CD8+ T-cells [76]. In this study, CD8+ T-cells were isolated from PBMCs of healthy donors and co-cultured with the SGC7901 gastric cancer cell line at a 5:1 ratio. Intriguingly, the results initially showed that tumor cells were capable to inhibit T-cell metabolism and this effect could be reversed by addition of glucose in the culture medium. The authors also demonstrated that co-culturing CD8+ T-cells with tumor cells enhanced the expression of TIGIT on their cellular surface. Interestingly, the blockade of TIGIT antigen with an anti-TIGIT antibody increased CD8+ T-cell metabolism, glucose consumption, as well as lactate production. It also restored T-cell effector functions by reversing gastric cancer cell-mediated inhibition of IFNγ production. This study thus highlights the potential of targeting TIGIT immune checkpoint to restore immune T-cells’ anti-tumor functions [76]. 

Finally, assessing the interaction of M1 macrophages with tumor cells can also be performed through direct co-culturing experiments [77]. To that end, co-cultures were carried out with RAW264.7 murine macrophage cells, which were previously polarized as M1 macrophage by LPS and INFγ treatment and the 4T1 breast cancer murine cell line. The latter cells were also primarily labeled with the CFSE fluorescent probe. Through a very detailed protocol, the authors described in their manuscript a clever method permitting visualizing 4T1 cells’ engulfment by macrophages which could be used for further immunotherapy studies aiming at targeting this specific subtype of immune cells [77]. 

Together, these data show that co-cultures involving direct cell-to-cell contact are becoming a widely used method for evaluating immunotherapies’ efficacy and the underlying molecular mechanisms associated with it. As discussed earlier, it has the advantage of being easy to set up and to give quick results. However, the experimental conditions should be carefully defined for not misinterpreting or over-interpreting the data obtained. Moreover, one of the major drawbacks of these experimental models is the lack of predictive value in terms of tumor heterogeneity, complexity, and 3D organization.

### 2.2. 3-Dimensional Methods

The major limitation for the development of new therapies acting on the TME, including immunotherapies, is the lack of consistent in vitro models. The traditional two-dimensional (2D) cancer models are still in use to study molecular and cellular features of tumors and sensitivity to treatments. However, many drawbacks have been identified for those models [78,79]. 2D culture methods indeed poorly represent patient’s tumor complexity, thus limiting their reliability. This is particularly true for solid tumors for which 3-dimensional organization is an important characteristic affecting their biological properties and survival capacities. 2D cell line cultures are indeed unable to fully reproduce tumor features such as microenvironment, immune system interaction, stromal compartment, and heterogeneity of cancer cells [80,81]. These limitations led, in the past years, to tremendous efforts for developing and designing new models capable of reproducing 3D tumor structuring, thus giving a rational intermediate between in vitro 2D culture and in vivo animal models.

#### 2.2.1. Spheroids

**Figure 2:**
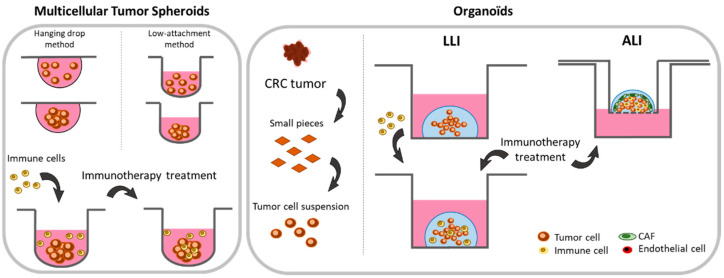
Schematic representation of 3-dimensional (3D) cultures with cell lines (spheroids) or patient tumor tissues (organoids) helpful for studying immunotherapies. The growth of tumor cell lines in 3D allows the formation of spheroids characterized by a necrotic core and a proliferative and metabolic gradient mimicking the 3D structure of a tumor. The spheroid allows the easy assessment of immune cells infiltration and the evaluation of strategies with pro-immunogenic potential. The main limitation of this model is the lack of heterogeneity related to the use of cell lines. 3D models made from small pieces of tumor tissues, also called organoids, have shown their ability to mimic tumor heterogeneity in terms of cellular components, TME, or tumor histology. Co-cultures of 3D-cells isolated from tumor tissues with immune cells in the liquid–liquid interface (LLI) method allow immune cell infiltration, activation, and their associated anti-tumor effect to be studied in a context closely reproducing tumor complexity, heterogeneity, and histology. On the other hand, the air–liquid interface (ALI) culture method has been developed to preserve the micro-environmental cellular components to further improve studies on immunotherapies in a context as close as possible than those observed in clinical solid tumors.

The easiest method to switch from 2D to 3D models is culturing spheroids, also called multicellular tumor spheroids (MCTS). These 3D structures can be easily obtained by incubating cell lines in low attachment plate. This specific surface favors cell-to-cell interactions, thus promoting spontaneous homotypic aggregation [82,83]. An alternative hanging drop method can also be used to generate spherical cell growth. Here, a drop of cell suspension is placed on a dish lid and then inverted onto the bottom chamber. Cells in the drop then aggregate and form spheroids (Figure 2) [84,85,86]. Importantly, it should be noted that not all cell lines are capable of assembling themselves to a 3D spheroid structure [87]. However, even if spheroids do not contain all the cell types and soluble factors that are present in the tumor microenvironment, they allow by their 3D organization mimicking cell-to-cell interactions, hypoxic conditions and low nutrient concentrations that otherwise characterize tumors. Moreover, it establishes a phenotypic heterogeneity which is not, or is poorly, observed in 2D cultures. Many studies have indeed highlighted a metabolic (oxygen consumption and lactate production) and proliferative gradient between the core and the periphery of the spheroids [88,89,90]. A necrotic part is also observed in the center of the spheroid as it is in vivo for solid tumors [85,91]. Another point which is important to consider is the sensitivity of cellular models to anti-cancer drugs. Indeed, many drugs show anti-cancer activity in 2D cellular models but their observed effects often do not predict activity in patients [79,92]. The 3D culture of spheroids, by more closely mimicking tumor complexity, has thus unraveled resistance mechanisms not found in a 2D cellular context [79,93,94,95]. Moreover, cell lines are able to form spheroids of varying density and it is known that dense spheroids show higher chemoresistance [93]. Therefore, 3D spheroids allow, early in their development, for the assessment of the anti-cancer activity of compounds on cancer cell populations displaying phenotypically distinctive traits and exposed to either high or low concentrations of tested molecules [96].

Spheroids also became a powerful tool for studying immunotherapies. Immune cells are indeed able to infiltrate them and to exert their biological effects [97,98,99]. Moreover, 3D-co-culturing of tumor cells with immune cells and fibroblasts demonstrated the accumulation of cytokines, chemokines, extracellular matrix components, and metalloproteins in the TME [97]. Interestingly, Rebelo et al. recently studied the increased infiltration capacity of THP-1 macrophages or donor blood-derived macrophages into heterotypic spheroids composed of the NCI-H157 lung cancer cell line and cancer-associated fibroblasts (CAF). The authors also reported the polarization of the infiltrating macrophages towards the M2 phenotype [97]. Similar experiments were conducted with spheroids formed by the LS174T CRC cell line co-cultured with immortalized fibroblasts (MRC-5). In this model, the authors showed the capability of leukocytes and monocytes to efficiently infiltrate heterotypic spheroids while T-cell infiltrate was limited [98]. Such co-cultured heterotypic spheroids are also a useful tool to evaluate therapeutic strategies. In line with this, Alonso–Nocelo et al. performed 3D co-cultures of A549 lung cancer cells with Jurkat T-cells (ratio 1:1) and evaluated T-cell infiltration into A549 cell line spheroids [99]. Interestingly, they reported the establishment of an inflammatory and immunosuppressive environment mediated by the 3D structuring of cancer cell spheroids which was increased when Jurkat T-cells were co-cultured. This makes such models of particular interest for studying anti-tumor immunity and evaluating anti-tumor activity of drugs [99] This new paradigm can be exemplified by the recent work published by Courau et al. [100]. Spheroids were grown with either HT29 or DLD-1 CRC cell lines and PBMCs added to the culture medium (ratio 1:10). This study first revealed the capacity of T- and NK-cells to infiltrate the spheroid and to be activated in the presence of IL-15, thus leading to spheroid destruction. Second, and maybe more importantly, this work identified the potential of using immunomodulatory antibodies targeting NKG2D ligands, a central activator of the NK cytotoxic response. The authors indeed observed an increased NK-cell infiltration and expression of the CD137 activation marker at their surface as well as a decreased expression of the CD16 receptor. Together, those results highlighted a NK-mediated anti-tumor response against CRC spheroids [100]. 

In addition to their usefulness and relevance for cancer research, 3D culturing approaches also permit animal uses to be reduced and should be considered as an alternative to them. Their ability to closely mimic crosstalks between immune, stromal (mostly fibroblasts), and tumor cells offers a good template to initially assess strategies aiming at targeting the TME [97]. Obviously, this method, which is easily available and of relatively low cost, also has some limitations. In particular, it does not permit the tumor structure and heterogeneity as well as its complex microenvironment to be completely represented. In addition, because they are often transformed or genetically modified, the cell lines used to form spheroids lack predictive power. However, to work around these limits, new methodologies such as organoids are now using patient-derived tumor slices to preserve the heterogeneous nature of tumors.

#### 2.2.2. Organoids

The organoid model is a 3-dimensional technology allowing the growth of a small-scale tissue in vitro, leading to its structure mimicking the in vivo parent organ [101,102]. Organoid culture is a promising approach to study the efficacy of immunotherapies in a context close to the patient’s physiology. The culture of the CRC organoid has long been studied and is now well characterized [103]. Particularly, it has been evidenced that organoids, developed from patient-derived colorectal tumor slices, allow the preservation of the tumor’s genetic heterogeneity, and, from a histological point of view, cells in the organoid are able to self-organize and to reproduce the morphological architecture of the original tumor. Hence, depending on the localization of the original surgical resection, organoids develop specific organizations reproducing the organ-like tissue [104,105,106].

In practice, organoids are established from small pieces of tumors isolated from surgical resections or biopsies. These fragments are crushed with an enzyme mix, filtered, and included into a Matrigel to subsequently be cultured in culture medium. A complete protocol has been designed by van de Wetering et al. [104]. With such approaches, development of co-cultures with immune cells can also be considered for immunotherapy development. The so-called liquid–liquid interface (LLI) method consists in separately culturing first organoids and immune cells before to establish co-cultures. The immune cells used in these studies can therefore be isolated either from healthy donors’ blood, and patients’ blood or tumors. Moreover, specific immune cell subpopulation (LT, NK, DC) can be cell sorted from the entire pool of PBMCs prior to co-culturing in order to study the response and effect of specific subpopulations on the organoids [68,107]. To initiate co-cultures, two distinctive approaches are usually considered. The first one consists of the digestion of organoids, addition of immune cells to the tumor suspension, and the regrowth of the organoid with immune cells. The second approach consists of the addition of immune cells directly to the culture medium without prior digestion of the organoids (Figure 2) [108].

The importance of organoids in cancer research can be exemplified by the work of Gonzales–Exposito et al. [109]. Co-cultures were performed with patient-derived CRC organoids and CD8+ T-cells isolated from PBMC of healthy donors (added 24 h after organoid formation). Suspensions were then treated with cibisatamab, a bispecific antibody recognizing CD3+ T-cells as well as the carcinoembryonic antigen (CEA) which is overexpressed by CRC cells. In this setting, the authors showed that tumors strongly expressing the CEA antigen responded to cibisatamab treatment while those expressing low CEA levels did not. Importantly, this study demonstrated the ability to redirect T-cells’ response against tumor cells and the potential of using organoids co-cultured with allogeneic immune cells [109]. To avoid non-specific allogeneic response of immune cells against “non-self” organoids, proper controls should, however, be used in this setting to ensure correct interpretation. To work around this problem, the co-culture of dMMR CRC organoids with PBMC isolated from the same patient can also be performed [110]. In the presence of IL-2- and CD28-coated antibodies, as well as by targeting PD-1, the authors succeeded in enriching the tumor-reactive T lymphocytes fraction and showed that reactive T-cells which were generated and were capable of effectively killing organoids [110]. This study also demonstrated that organoids express antigens permitting the recruitment, proliferation, and activation of T-cells.

To go on with the improvement of organoids, several research groups developed methods for culturing organoids in vitro in conditions favoring the maintenance of the whole microenvironment cell components. The so-called “air–liquid interface” (ALI) model consists of culturing minced tumor biopsy fragments that contain the entire cell populations actually present in the tumor (endogenous immune cells, fibroblasts, endothelial cells, epithelial cells, tumor cells) [111,112]. To do so, cells are embedded in a Matrigel solution and placed in an insert that was pre-coated with Matrigel. The insert is then positioned in a well containing the appropriate culture medium (Figure 2) [68]. This original approach helps mimic the intestinal membrane consisting of a monolayer of polarized epithelial cells with an apical surface towards the lumen and a basal surface towards the lamina propria [113]. An important issue for studying immune responses is the particular composition of TILs within the tumor microenvironment as it can generate an immunosuppressive environment promoting tumor progression. Importantly, it was reported that organoids prepared in such conditions can maintain the expression of the CD45 surface marker on leukocytes for 8 days. In the same study, the authors however noted a loss of CD3+ T-lymphocytes [113]. More recently, an alternative ALI culture method was developed in order to preserve TILs and the original tumor T-cell receptors for up to 30 days [114]. Therefore, co-culturing ALI-prepared organoids with infiltrating leukocytes now allows short-term studies for assessing the response to immunotherapies in the in vitro 3D model still harboring their original immune microenvironment. Importantly, after treatment, the cells that are present in the organoid can be harvested and analyzed (qRT-PCR, imaging, or flow cytometry) to study the efficacy of the therapy and to understand the underlying molecular mechanisms. By using this model, Neal et al. prepared organoids from murine tumors which were established in syngeneic models. Then, the authors treated the organoids for 7 days with immune checkpoint inhibitors (targeting PD-1 and PD-L1) and demonstrated by flow cytometry an increased number of CD8+ cytotoxic T-cells among the whole set of CD3+ T-cells as well as an increased cellular death evidenced by Annexin V/7-AAD double labeling. Finally, by performing RT-qPCR, the authors identified genes involved in this stimulation [114]. 

The ability to co-culture organoids with either heterologous or autologous PBMC as well as tumor resident immune cells is paving the way for studies aiming at characterizing in vitro the effect of the immune components on heterogeneous tumor models closely related to what can be found in vivo. These methodological developments open new perspectives in terms of testing drugs on immune cells as well as on their recruitment and/or activation. Moreover, the easy access to the tumor-like structure facilitates the understanding of the underlying molecular mechanisms and the identification of novel therapeutic opportunities. Importantly, organoids can be cryopreserved, allowing the development of tumoroid biobanks. Such models are of particular interest notably for screening molecules [104]. Finally, the development of the two LLI and ALI approaches offers two distinctive methodological options depending on the purpose of the experiments. If the objectives of the study are identifying molecules capable of attracting and/or activating peripheral immune cells or a subset of them to the tumor, the LLI approach seems to be the most appropriate. On the contrary, the ALI approach should be used for testing in vitro drugs and screening molecules capable of acting on the intrinsic immune cells and/or the immunosuppressive microenvironment. As a patient-derived xenograft, ALI organoids can also be thought of as a tool for personalized medicine. Organoids can therefore be considered as an excellent pre-clinical model bringing patients into basic cancer research and facilitating the transfer of knowledge into the clinical practice.

## 3. In Vivo CRC Models for Immunotherapy Studies

Even if organoids brought new options for drug discovery, in vitro models usually remain an initial step in the development of novel immunotherapies. Validation in animal models is indeed required to gain access to the whole parameters involved in the anti-tumor response such as pharmacokinetics, metabolism, immunity, or organ toxicity. It is therefore important to use animal models before translating new findings to humans. For most anti-cancer therapies, immunodeficient mice xenografted with human cancer cell lines are used. However, due to the lack of immune system, those models cannot apply to immunotherapy and therefore specific models should be considered.

### 3.1. Syngeneic Models

**Figure 3:**
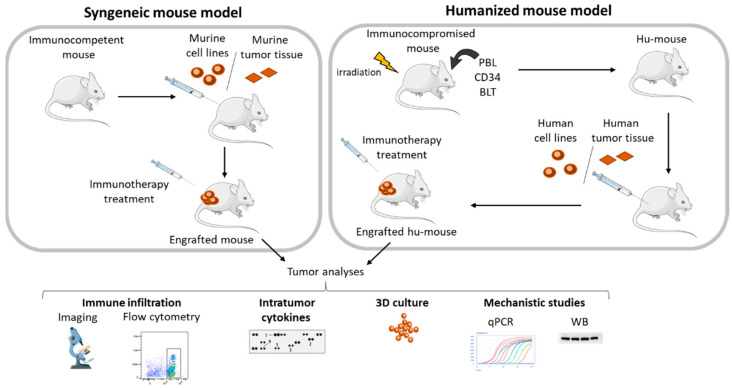
Mouse models used for immunotherapy research. The use of in vivo animal models is crucial for studying anti-tumor molecules acting on the TME. For immunotherapies, the easiest and most efficient model is the so-called syngeneic mouse model. A murine cancer cell line is injected into an immunocompetent mouse and the anti-cancer activity of the molecule of interest assessed through tumor growth inhibition, immune cell infiltration, and activation. Humanized mice models allow the efficacy of immunotherapies used in clinic on mice expressing human immune cells to be studied. Mice are humanized by injecting PBMC (PBL model), hematopoietic stem cells (CD34 model) or hematopoietic stem cells with grafting of human fetal liver and thymus (BLT model). These humanized mice are then grafted with either human tumor cell lines or human tumors samples (patient-derived xenograft). As for syngeneic models, tumors can be harvested and analyzed ex vivo for measuring immune infiltrate, cytokine release, and performed mechanistic studies. PBMC: Peripheral Blood Mononuclear Cell, PBL: Peripheral Blood Leukocyte, BLT: Bone marrow, Liver, Thymus.

The in vivo model the most commonly used by research groups working on immunotherapy is the syngeneic mouse model. This model consists of engraft murine cell lines previously grown in vitro into immunocompetent BALB/c or C57BL/6 mice. These murine models have an effective immune system. Hence, their treatment with immunotherapies allows treatment efficacy in terms of immune system activation and/or cytotoxic activity against tumors to be investigated (Figure 3) [29,67,115]. The major advantage of working with engrafted mouse cell lines is that the effect of the molecule can be easily determined by measuring the size of tumors. At the end of the procedure, tumors can be harvested, and several biological parameters possibly influenced by the treatment monitored. Immune cell infiltration can thus be evaluated by flow cytometry or immunohistochemistry. Expression of intra-tumor cytokines can be evaluated by cytokine array. Western blotting or RT-qPCR can also be performed to assess post-translational modifications or protein/genes expression levels. The whole set of data thus generated then improves our understanding of the mechanisms involved in either sensitivity or resistance to therapeutic interventions (Figure 3) [116,117,118,119,120]. Another major advantage of using syngeneic models is that engrafted cells can easily be manipulated and genetically modified in vitro prior to inoculation. Specific genes can be turned off to assess their impact on therapy and thus better ascribe the mechanism of action [121,122,123]. Genes encoding luciferase can also be introduced in the genome of the tumor cells to monitor their outcome in living organisms [124,125].

For CRC, the most commonly used syngeneic mouse models are the two CT26 and MC38 murine cell lines inoculated in immunocompetent BALB/c and C57BL/6 mouse, respectively [126]. The CT26 cell line is derived from a colon tumor formed in BALB/c mice exposed to N-nitroso-N-methylurethane, while MC38 cells were isolated from a colon tumor formed in a C57BL/6 mouse exposed to 1,2-dimethylhydrazine dihydrochloride (DMH). In human CRC, the majority of tumors present mutations in APC, KRAS, and TP53 genes. However, CT26 cells only have identified mutations in KRAS (G12D, V8M) but not in either APC or TP53 genes. Similarly, MC38 cells are mutated in the TP53 gene (G242V, S2581) but not in either KRAS or APC genes [127]. CT26 cells are pMMR and express CMH class I with a robust binding capability, but not CMH class II antigen presentation molecules. This model is often considered as the most immunogenic syngeneic mouse model and is described as a good responsive model for immunotherapeutic research [127,128]. In particular, CT26 cells show a high mutational load in contrast to most pMMR human tumor. Moreover, a large NK-cell infiltrate is observed in CT26 syngeneic models, a characteristic which does not mirror human colon tumors known to be poorly infiltrated by NK-cells [127,129]. On the other hand, the MC38 cell line is defined as an MSI model of CRC and present the highest mutational load among the ten most commonly used syngeneic mouse models evaluated to date [127]. Several groups used these models to evaluate CRC responses to classical immunotherapeutic agents. In a detailed study made on commonly used syngeneic models for different cancer location, CT26 tumor growth was significantly inhibited by both anti-CTLA4 and anti-PD-1 antibodies. In contrast, no anti-tumor activity was detected against the MC38 cells [130]. However, in another study, a complete inhibition of the MC38 tumor growth was observed after treatment with an anti-PD-L1 antibody [123].

In addition to the direct evaluation of immunotherapies, several research groups are interested in studying drug combinations capable of enhancing immune-directed molecule activity. For CRC, a strong interest is focused on combining oxaliplatin with molecules in order to potentiate its action. Oxaliplatin is indeed an effective chemotherapy used for treating CRC patients in clinics but its effect is often limited by the development of resistance [131,132]. Interestingly, oxaliplatin is also known to strongly induce ICD which favors the establishment of an immune-favorable microenvironment, thus facilitating the recruitment and activation of immune cells [133,134]. In a recent work, to overcome oxaliplatin resistance, this platinated compound was combined with an inhibitor of the ataxia telangiectasia and Rad3-related protein (ATR) kinase. The results first showed a strong in vitro synergistic effect in six different human colorectal cancer cell lines and their oxaliplatin-resistant counterparts. Importantly, this combination was also evaluated in the MC38 syngeneic mouse model. In this setting, a synergistic effect was also demonstrated in terms of tumor growth. In addition, the use of the immunocompetent model permitted further insights in the in vivo activity of this combination to be gained. Indeed, the authors clearly demonstrated that the combination of oxaliplatin with VE-822 (an ATR inhibitor) promoted an anti-tumor T-cell response which was characterized by an increased number of MC38-targeting IFNγ-producing CD8+ T-cells in mice that received the combined treatment compared to those treated with oxaliplatin alone [135]. Another interesting study investigated the effects of combining oxaliplatin with the anti-PD-L1 immune checkpoint inhibitor in the CT26 syngeneic mouse model. In this work, the authors successfully demonstrated the interest of combining these two classes of molecules to inhibit tumor growth. Maybe more importantly, this work clearly showed that combining oxaliplatin with an anti-PD-L1 monoclonal antibody led to an increased tumor infiltration of CD8+ T-cells, especially when the anti-PD-L1 molecule was injected before oxaliplatin. This study thus highlights the interest of these murine models to evaluate novel drug combinations and to optimize drug administration scheduling [136]. In agreement, the CT26 syngeneic mouse model was also used to evaluate the combination of MEK inhibitors with anti-PD-L1 monoclonal antibodies [29]. As discussed above, CT26 cells are cancer cells bearing activating mutations in the Ras pathway. However, beside the direct effect of MEK inhibition on cancer cells, the authors showed that G-38963 (which is similar to the MEK inhibitor Cobimetinib) can increase the number of effector-phenotype antigen-specific CD8+ T-cells within the tumor and act on the tumor-infiltrating CD8+ T-cells’ biology and survival. Importantly, the authors also reported that combining MEK inhibition with an anti-PD-L1 monoclonal antibody resulted in a synergistic and durable tumor response in mice [29]. However, in clinics, the effect of combining atezolizumab, a humanized IgG1 monoclonal antibody selectively targeting PD-L1, with Cobimetinib did not show any significant improvement of the overall survival of heavily pretreated pMMR mCRC patients compared with regorafenib or atezolizumab alone [137]. The discrepancy between preclinical and clinical data might be partly explained by the heterogeneous nature of mCRC at the advanced stages of the disease. This is particularly true for third-line-treated CRC patients who present otherwise chemo-refractory metastatic tumors. Moreover, the IMblaze370 clinical trial was not initially designed to assess the activity of the combination in different subgroups of patients. In addition, the CT26 cell line is known to be mutated in KRAS as well as for presenting MAPK1 and MET loci amplification [128]. This specific feature might therefore also impact their global response to MEK inhibitors. Finally, and as discussed above, the known immune responsiveness of the mouse CT26 syngeneic model is likely not to mirror the immune phenotype found in advanced pMMR tumors. These results underline the difficulties to translate preclinical data obtained on single cell type homogeneous tumors displaying genetic and immune-specific features to advanced clinical settings with inter- and intra-patient heterogeneous diseases. However, even if translation to humans might be tricky in the absence of biomarker assessments, the use of syngeneic mouse models remains a useful tool for deciphering clinical observations. This can be exemplified by the recent work demonstrating how liver metastasis might impact immunotherapy efficacy in patients with cancer [138]. In this work, the authors demonstrated that mice bearing subcutaneous syngeneic MC38 CRC tumors efficiently responded to anti-PD-L1 therapy while the same tumors failed to respond to this therapy in the presence of liver metastases. This phenomenon was related to a systemic loss of antigen-specific T-cells in mice bearing liver metastasis due an altered liver immune microenvironment favoring T-cell apoptosis. This model mirrors the systemic T-cell loss and decreased immunotherapy efficacy observed in patients with liver metastases. Importantly, this study also demonstrated that radiotherapy might reshape the liver immune microenvironment and abolish immunotherapy resistance. Together, these results suggest that liver metastases could serve as a potential biomarker for predicting immunotherapy response. Finally, the development of novel syngeneic mouse models, capable of better recapitulating the genetic origin of human CRC, might also prove useful for dealing with “immune desert” CRC. By crossing mice, mutated in four genes involved in colorectal cancer (APC, KRAS, TGFBR2, PTR53), Tauriello et al. established mutant mice developing pMMR metastatic intestinal tumors. From these tumors, the authors prepared organoids and engrafted them into immunocompetent mice. Interestingly, only a limited effect of anti-PD-L1 therapy could be observed on these MSS tumors. In contrast, TGF-β inhibition induced a significant reduction of the tumor mass thanks to the induction of a strong anti-tumor cytotoxic T-cell response. Moreover, TGF-β inhibition also prevented the formation of distant metastases and improved the response to anti-PD-L1 monoclonal antibody. Hence, the authors identified the TGF-β signaling as an interesting target for developing new strategies aimed at treating pMMR mCRC tumors that are otherwise resistant to immunotherapies [28].

Another important impact of mouse models in the field of immunotherapies is the easy access researchers have to immunodeficient as well as immunocompetent mice from the same origin. Indeed, by comparing the effects of molecules in mice with the same genetic background but in which the immune system is active or compromised, it is possible to evaluate the impact of the immune system has on the response to novel therapeutics or strategies. This approach was used for example to demonstrate that caloric restriction or hydroxycitrate improved the therapeutic outcome in CT26 CRC treated by chemotherapeutics in a T-cell-dependent fashion [139]. This strategy was also employed to explore the role of T-cells in the response to a novel immunotherapy targeting the phagocytic CD47 immune checkpoint [140]. There, the authors first demonstrated the anti-tumor activity of an anti-CD47 antibody in the treatment of A20 B lymphoma and MC38 CRC cells inoculated into wild-type BALB/c and C57BL/6 mice, respectively. However, when they looked at the efficacy of the treatment in immunocompromised nude mice, no effect could be observed on tumor growth, thus underlying the need for a T-cell-competent immune system in this process. Further experiments allowed the authors to demonstrate that the anti-cancer activity of the anti-CD47 antibody actually relied on DC cross-priming of CD8+ T-cells [140]. Finally, this approach can favor the emergence of new drug combinations which can be exemplified by the development of the prostaglandin E2 receptor 4 inhibitor, called TP-16 [141]. This molecule reduces the immunosuppressive myeloid cell functions. In this work, the authors first treated CT26 and MC38 CRC syngeneic tumor models with TP-16 and showed a significant reduction of the tumor mass. However, this effect was completely lost when the CRC cell lines were engrafted in immunocompromised nude mice. Again, these results stressed on the important role of having an intact immune system for observing an activity of this new molecule. Using in vitro approaches, the authors demonstrated that TP-16 reverses the immunosuppressive functions of PGE2 leading to an increased proportion of M1 macrophages, and a decreased fraction of M2 macrophages leading to a diminution of myeloid-derived suppressor cells thus favoring T-cell proliferation. These observations led the authors to combine TP-16 with an anti-PD-1 antibody in an immunocompetent CT26 syngeneic mouse model. Importantly, the combination showed a much more potent anti-tumor activity than either drug alone. The authors also reported that the combined effect of both compounds led to a more immune-favorable tumor microenvironment [141].

Together, these studies highlight the need of using syngeneic murine models for determining not only the efficacy of immunotherapies but also their underlying mechanism of action as well as the immune cells populations involved in. For colorectal cancer, syngeneic mouse models have yet proven to be a powerful tool for evaluating immunotherapies as well as strategies capable of turning cold tumors into hot responsive ones.

### 3.2. Humanized Mouse Model

One of the major limitations of syngeneic murine models is by definition the presence of a murine immune system which does not fully recapitulate the human one. Phenotypic and functional differences indeed exist between the two immune systems, thus leading to potential failures when findings are translated to the clinic [142]. Moreover, monoclonal antibodies used for targeting ICI in mice or in humans should be duplicated in order to target proteins in each species.

To circumvent this problem, humanized mice (hu-mice) were developed. They are models of immunocompromised mice displaying a reconstituted human immune system. The use of such models is a good alternative for testing the efficacy of immunotherapies on mice bearing human cell xenografts (Figure 3) [143,144]. Different strains of immunocompromised mice with deficiencies in specific immune cells populations are available to date: (i) Nude mice (Foxn1 mutated) have no T-cells, (ii) scid mice (severe combined immunodeficient) have no T- or B-cells, (iii) NOD-scid mice (non-obese diabetic severe combined immunodeficiency) have reduced NK-cell and myeloid cell functions, express human SIRP-α, and have no C5 complement, (iv) NSG (NOD/SCID/IL2R^null^) and NOG (NOD/SCID/IL2R^partial deficiency^) mice have no T-, B-, or NK-cells and show a reduced myeloid cell functions and no complement, (v) NRG mice (NOD/RAG1^null^/IL2R^null^) have no T-, B-, or NK-cells as well as no complement or myeloid cells, and (vi) BRG mice (BALB/c/RAG2^null^/IL2R^null^) have no T-, B-, or NK-cells, and no complement but, when compared to the NSG model, display more functional myeloid cells [145,146]. 

Mice humanization can be performed following different procedures. PMBCs (Peripheral Blood Leukocyte or PBL model) or hematopoietic stem cells (HSC) isolated from cord blood, bone marrow, mobilized peripheral human blood, or fetal liver (CD34 model) can be injected in immunocompromised mice. Alternatively, transplanting human tissues (thymus + liver) in immunodeficient mice (BLT model) can be done (Figure 3). The PBL model is probably the easiest to establish since it relies on a simple intra-splenic, intraperitoneal, or intravenous injection of human PBMC and it allows an efficient engraftment of T-cells [147]. However, the engraftment of myeloid cells is generally low and mice survival is relatively short because of the occurrence of graft-versus-host disease (GvHD) [148,149]. On the other hand, the CD34 model implies intra-femoral or intravenous injection of HSC and allows the development of multiple hematopoietic lineages and insures primary immune responses. However, the education of T-cells which occurs in the murine thymus is achieved by the murine major histocompatibility complex (MHC), thus preventing the development of human MHC T-cells [147]. To overcome this problem, it is now possible to graft fragments of the human fetal liver and thymus under the kidney capsule of the mouse in addition to intravenously injecting human CD34+ HSC (BLT method). Hence, the education of T-cells is taking place in human thymus and it allows for the development of multiple hematopoietic lineages and functional human MHC T-cells [147]. Nonetheless, this technique requires complex surgery and depends on the availability of human fetal tissue. Moreover, these mice are often subject to lethality because of xenogeneic GvHD [150,151].

Despite these limitations, those models can help to better understand what might happen in the human context. This can be illustrated by the work of Wang et al. in which irradiated NSG mice were humanized by injecting HSC isolated from fetal liver [152]. The authors first confirmed the humanization by labeling human CD45+ cells in the blood of the mice. Then, they implanted a human breast cancer cell line (MDA-MB-231) into the mammary fat pad and assessed the treatment efficacy of pembrolizumab, an anti-PD-1 monoclonal antibody used in clinics, on those humanized mice. Interestingly, while a significant reduction of the tumor mass was observed, the authors could not evidence any increase of CD45+, CD3+/CD4+, and CD3+/CD8+ infiltrating cells in the whole tumor. However, they demonstrated that CD8+ T-cells were relocalized from the tumor burden to the center after treatment with pembrolizumab. By depleting the human-derived CD8+ T-cells with an anti-human CD8+ antibody, the authors confirmed in hu-mice the absolute requirement of a competent CD8+ T-cell population for observing a cytotoxic effect of pembrolizumab. The anti-PD-1 monoclonal antibody indeed showed no activity on humanized mice when they were depleted of their hu-CD8+ T-cells [152]. Hu-mice are also a powerful tool to evaluate the activity of immune cells expressing a chimeric antigen receptor. In that sense, we can cite the work of Klichinsky et al. who generated chimeric antigen receptor macrophages (CAR-M) and tested their anti-cancer activity in the Hu-NSGS (NOD/SCID/IL2R^null^/hIL3/hGMCSF/hSF) mice model obtained after intravenous injection of human HSC [153]. The authors engrafted the human ovarian cancer SKOV3 cell line subcutaneously and then started intra-tumor injections of either CAR-M or unmodified macrophages. After 5 days of treatment, tumors were harvested, and RNA sequencing performed. The results clearly demonstrated the establishment of a pro-inflammatory tumor microenvironment promoting anti-tumor immunity when the hu-mice were treated with CAR-M [153].

Today, human cancer cell lines are still mostly used to engraft hu-mice. However, those cellular models are known to not strictly represent the tumor complexity and heterogeneity found in human cancers. Therefore, to better model the human pathology, humanized mice can be engrafted with patient-derived tumor tissue, the so-called patient-derived xenografts (PDX) (Figure 3) [154]. For CRC, PDX have been widely studied and have demonstrated high engraftment success rates [155,156]. PDX are isolated from patient tumor samples and transplanted subcutaneously in anaesthetized mice. The major advantage of PDXs is that they preserve the characteristics of the original tumor, both in terms of the gene expression profile and tumor heterogeneity [157,158]. Humanized mice bearing PDXs are therefore becoming a powerful tool for analyzing tumor and immune cell interaction and evaluating the efficacy of immunotherapies [159]. Recently, this approach was used to evaluate the response of CRC-PDX to Nivolumab, an anti-PD-1 monoclonal antibody used in clinics [160]. In this study, the authors first established their Hu-BRGS mice model by injecting into the facial vein or, when unsuccessful, into the liver CD34+ human HSC (isolated from cord blood) in irradiated BRGS (BALB/c/RAG2^null^/IL2R^null^/SIRPα^NOD^) mice. CRC-PDX were then established in nude mice in order to favor their initial growth and thereafter implanted in the Hu-CB-BRGS mice. When tumors volumes reached 150–300 mm^3^, treatments with Nivolumab were started. Strikingly, the authors demonstrated a significant tumor growth inhibition when the humanized mice were transplanted with CRC-PDX derived from MSI CRC patients while no efficacy could be observed in non-humanized immunodeficient BRGS mice. Moreover, the researchers could evidence, after treatment with Nivolumab, an increased infiltration of human TIL, T-cells, and CD8+ T-cells in PDX as well as an increased secretion of IFN-ɣ, thus demonstrating an anti-tumor immune response. Remarkably, when CRC-PDX derived from MSS CRC patients was used to engraft Hu-mice, the authors only showed a transient and partial anti-tumor effect of Nivolumab. This limited anti-cancer activity was accompanied by an absence of increased human TIL infiltration in the tumor [160]. This study thus recapitulated the observations made in the clinic, namely that dMMR CRC usually respond to ICI while pMMR CRC do not. This technological breakthrough opens new opportunities to better predict in patient-derived samples the clinical benefit of immunotherapies, but also to assess new strategies to overcome ICI resistance in CMS2-4 mCRC.

## 4. Conclusions

Our understanding of the impact of the immune system on the progression and prognostic of CRC led to tremendous efforts to better define tumor immune phenotypes. However, targeting most mCRC with immunotherapies remains to date challenging. Nonetheless, the development of novel experimental models led, in the last years, to the identification of some of the sources responsible for CRC non-responsiveness to immune-targeted therapies, thus identifying novel pharmacological opportunities to turn “cold” tumors into “hot” ones.

The better characterization of the intricate relationships existing between immune and tumor cells should, however, not limit our focus on these two unique compartments; the whole TME should indeed be considered. This assumption is particularly true for CRC if we consider that immunotherapies are, to date, mostly developed to treat metastatic diseases. However, nowadays, the characterization of metastasized CRC is still relatively poor, notably in terms of understanding of distant tissue TME [9]. In particular, the liver, which is one of the preferential location for CRC metastases, displays an immunosuppressive TME which likely facilitates CRC cells settlement but also shows specificities when compared to the original site of the tumor [9,161]. Accordingly, it has been reported that primary CRC tumors and CRC liver metastases diverged in terms of immune phenotypes [162]. Therefore, future successes in the field of CRC immunity are permitted due to the development of technologies allowing the evaluation of new innovative therapeutic strategies in this specific setting. In that sense, liver CRC metastasis-derived PDX established in humanized mice is likely to prove itself as a powerful tool to improve our understanding. However, the use of humanized mice remains complex, in particular for screening novel molecules or assessing innovative combinatorial strategies. Thus, emerging in vitro approaches should be designed to help circumventing the complexity of the in vivo models. In that sense, the development of multi-organoids-on-a-chip fluidic devices will prove useful for modeling primary and distant diseases as well as screening new drug modalities [163]. Development and implementation of novel microfluidics technologies might indeed provide solutions for managing small heterogeneous samples coming from patient-derived tumors or biopsy materials and for studying cancer cells’ behavior in a closed physiological context [164]. Interestingly, such a metastasis-on-a-chip fluidic system has yet to be set up to study early stages of human CRC metastasis [165]. A 3D microfluidic platform has also been developed for evaluating the migration of interferon-α-conditioned dendritic cells toward SW620 CRC cells [166]. However, while substantial progress has been made in developing chip modeling organ physiology, efforts for incorporating immune components in these micro-physiological systems have only been expanded recently [167]. In the future, such devices might be further improved by adding immune cells as well as tumoroids derived from either the primary or distant tissues originating from one single patient. Such a system will therefore permit simultaneous immunity to be assessed on both locations and to determine whether therapeutic strategies equally apply to both CRC tumors’ organ implantation sites.

## Figures and Tables

**Table 1:** Classification of 34 colorectal cancer (CRC) cell lines into consensus molecular subtype (CMS) subgroups. Human colorectal cancer cell lines are assigned into the best fitting CMS group according to Berg et al. [36].

CMS	Type	Colorectal Cell Lines
CMS1	MSI, Immune	Co115, DLD-1, HCT15, KM12, LoVo, SW48, Colo205, HCC2998
CMS2	Canonical	EB, FRI, IS3, LS1034, NCI-H508, SW116, SW1463, SW403, V9P
CMS3	Metabolic	CL-34, LS174T, CL-40, HT29, SW948, WiDr
CMS4	Mesenchymal	HCT116, RKO, TC71, CaCo2, CL-11, Colo678, IS1, SW480, SW837

## Data Availability

Not applicable.

## References

[1] Bray F., Ferlay J., Soerjomataram I., Siegel R.L., Torre L.A., Jemal A. (2018). Global Cancer Statistics 2018: GLOBOCAN Estimates of Incidence and Mortality Worldwide for 36 Cancers in 185 Countries. CA Cancer J. Clin..

[2] Koi M., Carethers J.M. (2017). The Colorectal Cancer Immune Microenvironment and Approach to Immunotherapies. Future Oncol..

[3] Siegel R.L., Miller K.D., Fuchs H.E., Jemal A. (2021). Cancer Statistics, 2021. CA Cancer J. Clin..

[4] Sobrero A., Lonardi S., Rosati G., Di Bartolomeo M., Ronzoni M., Pella N., Scartozzi M., Banzi M., Zampino M.G., Pasini F. (2018). FOLFOX or CAPOX in Stage II to III Colon Cancer: Efficacy Results of the Italian Three or Six Colon Adjuvant Trial. J. Clin. Oncol..

[5] André T., Meyerhardt J., Iveson T., Sobrero A., Yoshino T., Souglakos I., Grothey A., Niedzwiecki D., Saunders M., Labianca R. (2020). Effect of Duration of Adjuvant Chemotherapy for Patients with Stage III Colon Cancer (IDEA Collaboration): Final Results from a Prospective, Pooled Analysis of Six Randomised, Phase 3 Trials. Lancet Oncol..

[6] Van Cutsem E., Cervantes A., Adam R., Sobrero A., Van Krieken J.H., Aderka D., Aranda Aguilar E., Bardelli A., Benson A., Bodoky G. (2016). ESMO Consensus Guidelines for the Management of Patients with Metastatic Colorectal Cancer. Ann. Oncol..

[7] Pagès F., Berger A., Camus M., Sanchez-Cabo F., Costes A., Molidor R., Mlecnik B., Kirilovsky A., Nilsson M., Damotte D. (2005). Effector Memory T Cells, Early Metastasis, and Survival in Colorectal Cancer. N. Engl. J. Med..

[8] Galon J., Costes A., Sanchez-Cabo F., Kirilovsky A., Mlecnik B., Lagorce-Pagès C., Tosolini M., Camus M., Berger A., Wind P. (2006). Type, Density, and Location of Immune Cells within Human Colorectal Tumors Predict Clinical Outcome. Science.

[9] Roelands J., Kuppen P.J.K., Vermeulen L., Maccalli C., Decock J., Wang E., Marincola F.M., Bedognetti D., Hendrickx W. (2017). Immunogenomic Classification of Colorectal Cancer and Therapeutic Implications. Int. J. Mol. Sci..

[10] Galon J., Pagès F., Marincola F.M., Angell H.K., Thurin M., Lugli A., Zlobec I., Berger A., Bifulco C., Botti G. (2012). Cancer Classification Using the Immunoscore: A Worldwide Task Force. J. Transl. Med..

[11] Pagès F., Mlecnik B., Marliot F., Bindea G., Ou F.-S., Bifulco C., Lugli A., Zlobec I., Rau T.T., Berger M.D. (2018). International Validation of the Consensus Immunoscore for the Classification of Colon Cancer: A Prognostic and Accuracy Study. Lancet.

[12] Ciardiello D., Vitiello P.P., Cardone C., Martini G., Troiani T., Martinelli E., Ciardiello F. (2019). Immunotherapy of Colorectal Cancer: Challenges for Therapeutic Efficacy. Cancer Treat. Rev..

[13] Le D.T., Uram J.N., Wang H., Bartlett B.R., Kemberling H., Eyring A.D., Skora A.D., Luber B.S., Azad N.S., Laheru D. (2015). PD-1 Blockade in Tumors with Mismatch-Repair Deficiency. N. Engl. J. Med..

[14] Cohen R., Rousseau B., Vidal J., Colle R., Diaz L.A., André T. (2020). Immune Checkpoint Inhibition in Colorectal Cancer: Microsatellite Instability and Beyond. Target. Oncol..

[15] Marmorino F., Boccaccino A., Germani M.M., Falcone A., Cremolini C. (2020). Immune Checkpoint Inhibitors in PMMR Metastatic Colorectal Cancer: A Tough Challenge. Cancers.

[16] Jass J.R. (2007). Classification of Colorectal Cancer Based on Correlation of Clinical, Morphological and Molecular Features. Histopathology.

[17] Bindea G., Mlecnik B., Tosolini M., Kirilovsky A., Waldner M., Obenauf A.C., Angell H., Fredriksen T., Lafontaine L., Berger A. (2013). Spatiotemporal Dynamics of Intratumoral Immune Cells Reveal the Immune Landscape in Human Cancer. Immunity.

[18] McGranahan N., Furness A.J.S., Rosenthal R., Ramskov S., Lyngaa R., Saini S.K., Jamal-Hanjani M., Wilson G.A., Birkbak N.J., Hiley C.T. (2016). Clonal Neoantigens Elicit T Cell Immunoreactivity and Sensitivity to Immune Checkpoint Blockade. Science.

[19] Mouw K.W., Goldberg M.S., Konstantinopoulos P.A., D’Andrea A.D. (2017). DNA Damage and Repair Biomarkers of Immunotherapy Response. Cancer Discov..

[20] Germano G., Lamba S., Rospo G., Barault L., Magrì A., Maione F., Russo M., Crisafulli G., Bartolini A., Lerda G. (2017). Inactivation of DNA Repair Triggers Neoantigen Generation and Impairs Tumour Growth. Nature.

[21] Llosa N.J., Cruise M., Tam A., Wicks E.C., Hechenbleikner E.M., Taube J.M., Blosser R.L., Fan H., Wang H., Luber B.S. (2015). The Vigorous Immune Microenvironment of Microsatellite Instable Colon Cancer Is Balanced by Multiple Counter-Inhibitory Checkpoints. Cancer Discov..

[22] Becht E., de Reyniès A., Giraldo N.A., Pilati C., Buttard B., Lacroix L., Selves J., Sautès-Fridman C., Laurent-Puig P., Fridman W.H. (2016). Immune and Stromal Classification of Colorectal Cancer Is Associated with Molecular Subtypes and Relevant for Precision Immunotherapy. Clin. Cancer Res..

[23] Malesci A., Laghi L., Bianchi P., Delconte G., Randolph A., Torri V., Carnaghi C., Doci R., Rosati R., Montorsi M. (2007). Reduced Likelihood of Metastases in Patients with Microsatellite-Unstable Colorectal Cancer. Clin. Cancer Res..

[24] Boland C.R., Goel A. (2010). Microsatellite Instability in Colorectal Cancer. Gastroenterology.

[25] Guinney J., Dienstmann R., Wang X., de Reyniès A., Schlicker A., Soneson C., Marisa L., Roepman P., Nyamundanda G., Angelino P. (2015). The Consensus Molecular Subtypes of Colorectal Cancer. Nat. Med..

[26] Angelova M., Charoentong P., Hackl H., Fischer M.L., Snajder R., Krogsdam A.M., Waldner M.J., Bindea G., Mlecnik B., Galon J. (2015). Characterization of the Immunophenotypes and Antigenomes of Colorectal Cancers Reveals Distinct Tumor Escape Mechanisms and Novel Targets for Immunotherapy. Genome Biol..

[27] Courau T., Nehar-Belaid D., Florez L., Levacher B., Vazquez T., Brimaud F., Bellier B., Klatzmann D. (2016). TGF-β and VEGF Cooperatively Control the Immunotolerant Tumor Environment and the Efficacy of Cancer Immunotherapies. JCI Insight.

[28] Tauriello D.V.F., Palomo-Ponce S., Stork D., Berenguer-Llergo A., Badia-Ramentol J., Iglesias M., Sevillano M., Ibiza S., Cañellas A., Hernando-Momblona X. (2018). TGFβ Drives Immune Evasion in Genetically Reconstituted Colon Cancer Metastasis. Nature.

[29] Ebert P.J.R., Cheung J., Yang Y., McNamara E., Hong R., Moskalenko M., Gould S.E., Maecker H., Irving B.A., Kim J.M. (2016). MAP Kinase Inhibition Promotes T Cell and Anti-Tumor Activity in Combination with PD-L1 Checkpoint Blockade. Immunity.

[30] Kanterman J., Sade-Feldman M., Biton M., Ish-Shalom E., Lasry A., Goldshtein A., Hubert A., Baniyash M. (2014). Adverse Immunoregulatory Effects of 5FU and CPT11 Chemotherapy on Myeloid-Derived Suppressor Cells and Colorectal Cancer Outcomes. Cancer Res..

[31] Emens L.A., Middleton G. (2015). The Interplay of Immunotherapy and Chemotherapy: Harnessing Potential Synergies. Cancer Immunol. Res..

[32] Galluzzi L., Buqué A., Kepp O., Zitvogel L., Kroemer G. (2015). Immunological Effects of Conventional Chemotherapy and Targeted Anticancer Agents. Cancer Cell.

[33] Terme M., Pernot S., Marcheteau E., Sandoval F., Benhamouda N., Colussi O., Dubreuil O., Carpentier A.F., Tartour E., Taieb J. (2013). VEGFA-VEGFR Pathway Blockade Inhibits Tumor-Induced Regulatory T-Cell Proliferation in Colorectal Cancer. Cancer Res..

[34] Wang L., Wei Y., Fang W., Lu C., Chen J., Cui G., Diao H. (2017). Cetuximab Enhanced the Cytotoxic Activity of Immune Cells during Treatment of Colorectal Cancer. Cell. Physiol. Biochem..

[35] Raimondi C., Nicolazzo C., Gradilone A., Giannini G., De Falco E., Chimenti I., Varriale E., Hauch S., Plappert L., Cortesi E. (2014). Circulating Tumor Cells: Exploring Intratumor Heterogeneity of Colorectal Cancer. Cancer Biol. Ther..

[36] Berg K.C.G., Eide P.W., Eilertsen I.A., Johannessen B., Bruun J., Danielsen S.A., Bjørnslett M., Meza-Zepeda L.A., Eknæs M., Lind G.E. (2017). Multi-Omics of 34 Colorectal Cancer Cell Lines—A Resource for Biomedical Studies. Mol. Cancer.

[37] Sahin I.H., Akce M., Alese O., Shaib W., Lesinski G.B., El-Rayes B., Wu C. (2019). Immune Checkpoint Inhibitors for the Treatment of MSI-H/MMR-D Colorectal Cancer and a Perspective on Resistance Mechanisms. Br. J. Cancer.

[38] Gupta R., Sinha S., Paul R.N. (2018). The Impact of Microsatellite Stability Status in Colorectal Cancer. Curr. Probl. Cancer.

[39] Brandi J., Manfredi M., Speziali G., Gosetti F., Marengo E., Cecconi D. (2018). Proteomic Approaches to Decipher Cancer Cell Secretome. Semin. Cell Dev. Biol..

[40] Mohebbi B., Ashtibaghaei K., Hashemi M., Hashemi M., Asadzadeh Aghdaei H., Zali M.R. (2019). Conditioned Medium from Cultured Colorectal Cancer Cells Affects Peripheral Blood Mononuclear Cells Inflammatory Phenotype in Vitro. Iran. J. Med. Sci..

[41] Lee C., Jeong H., Bae Y., Shin K., Kang S., Kim H., Oh J., Bae H. (2019). Targeting of M2-like Tumor-Associated Macrophages with a Melittin-Based pro-Apoptotic Peptide. J. Immunother. Cancer.

[42] Di Mitri D., Mirenda M., Vasilevska J., Calcinotto A., Delaleu N., Revandkar A., Gil V., Boysen G., Losa M., Mosole S. (2019). Re-Education of Tumor-Associated Macrophages by CXCR2 Blockade Drives Senescence and Tumor Inhibition in Advanced Prostate Cancer. Cell Rep..

[43] Andón F.T., Digifico E., Maeda A., Erreni M., Mantovani A., Alonso M.J., Allavena P. (2017). Targeting Tumor Associated Macrophages: The New Challenge for Nanomedicine. Semin. Immunol..

[44] Grugan K.D., McCabe F.L., Kinder M., Greenplate A.R., Harman B.C., Ekert J.E., van Rooijen N., Anderson G.M., Nemeth J.A., Strohl W.R. (2012). Tumor-Associated Macrophages Promote Invasion While Retaining Fc-Dependent Anti-Tumor Function. J. Immunol..

[45] Solinas G., Schiarea S., Liguori M., Fabbri M., Pesce S., Zammataro L., Pasqualini F., Nebuloni M., Chiabrando C., Mantovani A. (2010). Tumor-Conditioned Macrophages Secrete Migration-Stimulating Factor: A New Marker for M2-Polarization, Influencing Tumor Cell Motility. J. Immunol..

[46] Benner B., Scarberry L., Suarez-Kelly L.P., Duggan M.C., Campbell A.R., Smith E., Lapurga G., Jiang K., Butchar J.P., Tridandapani S. (2019). Generation of Monocyte-Derived Tumor-Associated Macrophages Using Tumor-Conditioned Media Provides a Novel Method to Study Tumor-Associated Macrophages in Vitro. J. Immunother. Cancer.

[47] Dong H., Yang Y., Gao C., Sun H., Wang H., Hong C., Wang J., Gong F., Gao X. (2020). Lactoferrin-Containing Immunocomplex Mediates Antitumor Effects by Resetting Tumor-Associated Macrophages to M1 Phenotype. J. Immunother. Cancer.

[48] Sawa-Wejksza K., Dudek A., Lemieszek M., Kaławaj K., Kandefer-Szerszeń M. (2018). Colon Cancer-Derived Conditioned Medium Induces Differentiation of THP-1 Monocytes into a Mixed Population of M1/M2 Cells. Tumour Biol..

[49] Adil A.A.M., Vallinayagam L., Chitra K., Jamal S., Pandurangan A.K., Ahmed N. (2019). Increased Expression of TGF-β and IFN-γ in Peripheral Blood Mononuclear Cells (PBMCs) Cultured in Conditioned Medium (CM) of K562 Cell Culture. J. Environ. Pathol. Toxicol. Oncol..

[50] Teng L., Liu L., Su Y., Yuan X., Li J., Fu Q., Chen S., Wang C. (2011). Suppression of Alloimmunity in Mice by Regulatory T Cells Converted with Conditioned Media. J. Surg. Res..

[51] Wang D., Yang L., Yu W., Wu Q., Lian J., Li F., Liu S., Li A., He Z., Liu J. (2019). Colorectal Cancer Cell-Derived CCL20 Recruits Regulatory T Cells to Promote Chemoresistance via FOXO1/CEBPB/NF-ΚB Signaling. J. Immunother. Cancer.

[52] Heeran A.B., Dunne M.R., Morrissey M.E., Buckley C.E., Clarke N., Cannon A., Donlon N.E., Nugent T.S., Durand M., Dunne C. (2021). The Protein Secretome Is Altered in Rectal Cancer Tissue Compared to Normal Rectal Tissue, and Alterations in the Secretome Induce Enhanced Innate Immune Responses. Cancers.

[53] Madden E.C., Gorman A.M., Logue S.E., Samali A. (2020). Tumour Cell Secretome in Chemoresistance and Tumour Recurrence. Trends Cancer.

[54] Kather J.N., Halama N., Jaeger D. (2018). Genomics and Emerging Biomarkers for Immunotherapy of Colorectal Cancer. Semin. Cancer Biol..

[55] Slaney C.Y., Kershaw M.H., Darcy P.K. (2014). Trafficking of T Cells into Tumors. Cancer Res..

[56] Kmiecik J., Poli A., Brons N.H.C., Waha A., Eide G.E., Enger P.Ø., Zimmer J., Chekenya M. (2013). Elevated CD3+ and CD8+ Tumor-Infiltrating Immune Cells Correlate with Prolonged Survival in Glioblastoma Patients despite Integrated Immunosuppressive Mechanisms in the Tumor Microenvironment and at the Systemic Level. J. Neuroimmunol..

[57] Tyrer P.C., Bean E.G., Ruth Foxwell A., Pavli P. (2011). Effects of Bacterial Products on Enterocyte-Macrophage Interactions in Vitro. Biochem. Biophys. Res. Commun..

[58] Nagata M., Yamamoto H., Tabe K., Sakamoto Y. (2001). Eosinophil Transmigration across VCAM-1-Expressing Endothelial Cells Is Upregulated by Antigen-Stimulated Mononuclear Cells. Int. Arch. Allergy Immunol..

[59] Wang Y., Zhang H., He H., Ai K., Yu W., Xiao X., Qin Y., Zhang L., Xiong H., Zhou G. (2020). LRCH1 Suppresses Migration of CD4+ T Cells and Refers to Disease Activity in Ulcerative Colitis. Int. J. Med. Sci..

[60] Huang R.-P. (2007). An Array of Possibilities in Cancer Research Using Cytokine Antibody Arrays. Expert Rev. Proteom..

[61] Zhang Y., Du W., Chen Z., Xiang C. (2017). Upregulation of PD-L1 by SPP1 Mediates Macrophage Polarization and Facilitates Immune Escape in Lung Adenocarcinoma. Exp. Cell Res..

[62] Wang C.-J., Zhu C.-C., Xu J., Wang M., Zhao W.-Y., Liu Q., Zhao G., Zhang Z.-Z. (2019). The LncRNA UCA1 Promotes Proliferation, Migration, Immune Escape and Inhibits Apoptosis in Gastric Cancer by Sponging Anti-Tumor MiRNAs. Mol. Cancer.

[63] Harlin H., Meng Y., Peterson A.C., Zha Y., Tretiakova M., Slingluff C., McKee M., Gajewski T.F. (2009). Chemokine Expression in Melanoma Metastases Associated with CD8+ T-Cell Recruitment. Cancer Res..

[64] Wang X.-Q., Zhou W.-J., Luo X.-Z., Tao Y., Li D.-J. (2017). Synergistic Effect of Regulatory T Cells and Proinflammatory Cytokines in Angiogenesis in the Endometriotic Milieu. Hum. Reprod..

[65] Hennel R., Brix N., Seidl K., Ernst A., Scheithauer H., Belka C., Lauber K. (2014). Release of Monocyte Migration Signals by Breast Cancer Cell Lines after Ablative and Fractionated γ-Irradiation. Radiat. Oncol..

[66] Yu Y., Blokhuis B., Derks Y., Kumari S., Garssen J., Redegeld F. (2018). Human Mast Cells Promote Colon Cancer Growth via Bidirectional Crosstalk: Studies in 2D and 3D Coculture Models. Oncoimmunology.

[67] Liu C., Liu R., Wang B., Lian J., Yao Y., Sun H., Zhang C., Fang L., Guan X., Shi J. (2021). Blocking IL-17A Enhances Tumor Response to Anti-PD-1 Immunotherapy in Microsatellite Stable Colorectal Cancer. J. Immunother. Cancer.

[68] Grützkau A., Radbruch A. (2010). Small but Mighty: How the MACS-Technology Based on Nanosized Superparamagnetic Particles Has Helped to Analyze the Immune System within the Last 20 Years. Cytom. A.

[69] Rios F.J., Touyz R.M., Montezano A.C. (2017). Isolation and Differentiation of Human Macrophages. Methods Mol. Biol..

[70] Trickett A., Kwan Y.L. (2003). T Cell Stimulation and Expansion Using Anti-CD3/CD28 Beads. J. Immunol. Methods.

[71] Tomchuck S.L., Leung W.H., Dallas M.H. (2015). Enhanced Cytotoxic Function of Natural Killer and CD3+CD56+ Cells in Cord Blood after Culture. Biol. Blood Marrow Transplant..

[72] Vogel D.Y.S., Glim J.E., Stavenuiter A.W.D., Breur M., Heijnen P., Amor S., Dijkstra C.D., Beelen R.H.J. (2014). Human Macrophage Polarization in Vitro: Maturation and Activation Methods Compared. Immunobiology.

[73] Melief J., Wickström S., Kiessling R., Pico de Coaña Y. (2019). Assessment of Antitumor T-Cell Responses by Flow Cytometry After Coculture of Tumor Cells with Autologous Tumor-Infiltrating Lymphocytes. Methods Mol. Biol..

[74] Minute L., Teijeira A., Sanchez-Paulete A.R., Ochoa M.C., Alvarez M., Otano I., Etxeberrria I., Bolaños E., Azpilikueta A., Garasa S. (2020). Cellular Cytotoxicity Is a Form of Immunogenic Cell Death. J. Immunother. Cancer.

[75] Yu W., Wang Y., Guo P. (2018). Notch Signaling Pathway Dampens Tumor-Infiltrating CD8+ T Cells Activity in Patients with Colorectal Carcinoma. Biomed. Pharmacother. Biomed. Pharmacother..

[76] He W., Zhang H., Han F., Chen X., Lin R., Wang W., Qiu H., Zhuang Z., Liao Q., Zhang W. (2017). CD155T/TIGIT Signaling Regulates CD8+ T-Cell Metabolism and Promotes Tumor Progression in Human Gastric Cancer. Cancer Res..

[77] Zaidi N.E., Shazali N.A.H., Chor A.L.T., Osman M.A., Ibrahim K., Jaoi-Edward M., Afizan Nik Abd Rahman N.M. (2019). Time-Lapse 2D Imaging of Phagocytic Activity in M1 Macrophage-4T1 Mouse Mammary Carcinoma Cells in Co-Cultures. J. Vis. Exp. JoVE.

[78] Souza A.G., Silva I.B.B., Campos-Fernandez E., Barcelos L.S., Souza J.B., Marangoni K., Goulart L.R., Alonso-Goulart V. (2018). Comparative Assay of 2D and 3D Cell Culture Models: Proliferation, Gene Expression and Anticancer Drug Response. Curr. Pharm. Des..

[79] Tung Y.-C., Hsiao A.Y., Allen S.G., Torisawa Y., Ho M., Takayama S. (2011). High-Throughput 3D Spheroid Culture and Drug Testing Using a 384 Hanging Drop Array. Analyst.

[80] Breslin S., O’Driscoll L. (2013). Three-Dimensional Cell Culture: The Missing Link in Drug Discovery. Drug Discov. Today.

[81] Silva-Almeida C., Ewart M.-A., Wilde C. (2020). 3D Gastrointestinal Models and Organoids to Study Metabolism in Human Colon Cancer. Semin. Cell Dev. Biol..

[82] Vinci M., Gowan S., Boxall F., Patterson L., Zimmermann M., Court W., Lomas C., Mendiola M., Hardisson D., Eccles S.A. (2012). Advances in Establishment and Analysis of Three-Dimensional Tumor Spheroid-Based Functional Assays for Target Validation and Drug Evaluation. BMC Biol..

[83] Kim J.B. (2005). Three-Dimensional Tissue Culture Models in Cancer Biology. Semin. Cancer Biol..

[84] Foty R. (2011). A Simple Hanging Drop Cell Culture Protocol for Generation of 3D Spheroids. J. Vis. Exp. JoVE.

[85] Weiswald L.-B., Bellet D., Dangles-Marie V. (2015). Spherical Cancer Models in Tumor Biology. Neoplasia.

[86] Kelm J.M., Timmins N.E., Brown C.J., Fussenegger M., Nielsen L.K. (2003). Method for Generation of Homogeneous Multicellular Tumor Spheroids Applicable to a Wide Variety of Cell Types. Biotechnol. Bioeng..

[87] Friedrich J., Ebner R., Kunz-Schughart L.A. (2007). Experimental Anti-Tumor Therapy in 3-D: Spheroids--Old Hat or New Challenge?. Int. J. Radiat. Biol..

[88] Hamilton G., Rath B. (2019). Applicability of Tumor Spheroids for in Vitro Chemosensitivity Assays. Expert Opin. Drug Metab. Toxicol..

[89] Grimes D.R., Kelly C., Bloch K., Partridge M. (2014). A Method for Estimating the Oxygen Consumption Rate in Multicellular Tumour Spheroids. J. R. Soc. Interface.

[90] Gong X., Lin C., Cheng J., Su J., Zhao H., Liu T., Wen X., Zhao P. (2015). Generation of Multicellular Tumor Spheroids with Microwell-Based Agarose Scaffolds for Drug Testing. PLoS ONE.

[91] Dubessy C., Merlin J.M., Marchal C., Guillemin F. (2000). Spheroids in Radiobiology and Photodynamic Therapy. Crit. Rev. Oncol. Hematol..

[92] Mó I., Alves C.G., de Melo-Diogo D., Lima-Sousa R., Correia I.J. (2020). Assessing the Combinatorial Chemo-Photothermal Therapy Mediated by Sulfobetaine Methacrylate-Functionalized Nanoparticles in 2D and 3D In Vitro Cancer Models. Biotechnol. J..

[93] Imamura Y., Mukohara T., Shimono Y., Funakoshi Y., Chayahara N., Toyoda M., Kiyota N., Takao S., Kono S., Nakatsura T. (2015). Comparison of 2D- and 3D-Culture Models as Drug-Testing Platforms in Breast Cancer. Oncol. Rep..

[94] Nunes A.S., Costa E.C., Barros A.S., de Melo-Diogo D., Correia I.J. (2019). Establishment of 2D Cell Cultures Derived From 3D MCF-7 Spheroids Displaying a Doxorubicin Resistant Profile. Biotechnol. J..

[95] Azharuddin M., Roberg K., Dhara A.K., Jain M.V., Darcy P., Hinkula J., Slater N.K.H., Patra H.K. (2019). Dissecting Multi Drug Resistance in Head and Neck Cancer Cells Using Multicellular Tumor Spheroids. Sci. Rep..

[96] Minchinton A.I., Tannock I.F. (2006). Drug Penetration in Solid Tumours. Nat. Rev. Cancer.

[97] Rebelo S.P., Pinto C., Martins T.R., Harrer N., Estrada M.F., Loza-Alvarez P., Cabeçadas J., Alves P.M., Gualda E.J., Sommergruber W. (2018). 3D-3-Culture: A Tool to Unveil Macrophage Plasticity in the Tumour Microenvironment. Biomaterials.

[98] Herter S., Morra L., Schlenker R., Sulcova J., Fahrni L., Waldhauer I., Lehmann S., Reisländer T., Agarkova I., Kelm J.M. (2017). A Novel Three-Dimensional Heterotypic Spheroid Model for the Assessment of the Activity of Cancer Immunotherapy Agents. Cancer Immunol. Immunother..

[99] Alonso-Nocelo M., Abuín C., López-López R., de la Fuente M. (2016). Development and Characterization of a Three-Dimensional Co-Culture Model of Tumor T Cell Infiltration. Biofabrication.

[100] Courau T., Bonnereau J., Chicoteau J., Bottois H., Remark R., Assante Miranda L., Toubert A., Blery M., Aparicio T., Allez M. (2019). Cocultures of Human Colorectal Tumor Spheroids with Immune Cells Reveal the Therapeutic Potential of MICA/B and NKG2A Targeting for Cancer Treatment. J. Immunother. Cancer.

[101] Hickman J.A., Graeser R., de Hoogt R., Vidic S., Brito C., Gutekunst M., van der Kuip H., IMI PREDECT Consortium (2014). Three-Dimensional Models of Cancer for Pharmacology and Cancer Cell Biology: Capturing Tumor Complexity in Vitro/Ex Vivo. Biotechnol. J..

[102] Ji D.-B., Wu A.-W. (2020). Organoid in Colorectal Cancer: Progress and Challenges. Chin. Med. J..

[103] Li X., Larsson P., Ljuslinder I., Öhlund D., Myte R., Löfgren-Burström A., Zingmark C., Ling A., Edin S., Palmqvist R. (2020). Ex Vivo Organoid Cultures Reveal the Importance of the Tumor Microenvironment for Maintenance of Colorectal Cancer Stem Cells. Cancers.

[104] Van de Wetering M., Francies H.E., Francis J.M., Bounova G., Iorio F., Pronk A., van Houdt W., van Gorp J., Taylor-Weiner A., Kester L. (2015). Prospective Derivation of a Living Organoid Biobank of Colorectal Cancer Patients. Cell.

[105] Sasaki N., Clevers H. (2018). Studying Cellular Heterogeneity and Drug Sensitivity in Colorectal Cancer Using Organoid Technology. Curr. Opin. Genet. Dev..

[106] Roerink S.F., Sasaki N., Lee-Six H., Young M.D., Alexandrov L.B., Behjati S., Mitchell T.J., Grossmann S., Lightfoot H., Egan D.A. (2018). Intra-Tumour Diversification in Colorectal Cancer at the Single-Cell Level. Nature.

[107] Riedhammer C., Halbritter D., Weissert R. (2016). Peripheral Blood Mononuclear Cells: Isolation, Freezing, Thawing, and Culture. Methods Mol. Biol..

[108] Bar-Ephraim Y.E., Kretzschmar K., Clevers H. (2020). Organoids in Immunological Research. Nat. Rev. Immunol..

[109] Gonzalez-Exposito R., Semiannikova M., Griffiths B., Khan K., Barber L.J., Woolston A., Spain G., von Loga K., Challoner B., Patel R. (2019). CEA Expression Heterogeneity and Plasticity Confer Resistance to the CEA-Targeting Bispecific Immunotherapy Antibody Cibisatamab (CEA-TCB) in Patient-Derived Colorectal Cancer Organoids. J. Immunother. Cancer.

[110] Dijkstra K.K., Cattaneo C.M., Weeber F., Chalabi M., van de Haar J., Fanchi L.F., Slagter M., van der Velden D.L., Kaing S., Kelderman S. (2018). Generation of Tumor-Reactive T Cells by Co-Culture of Peripheral Blood Lymphocytes and Tumor Organoids. Cell.

[111] Usui T., Sakurai M., Umata K., Yamawaki H., Ohama T., Sato K. (2018). Preparation of Human Primary Colon Tissue-Derived Organoid Using Air Liquid Interface Culture. Curr. Protoc. Toxicol..

[112] Li X., Ootani A., Kuo C. (2016). An Air-Liquid Interface Culture System for 3D Organoid Culture of Diverse Primary Gastrointestinal Tissues. Methods Mol. Biol..

[113] Finnberg N.K., Gokare P., Lev A., Grivennikov S.I., MacFarlane A.W., Campbell K.S., Winters R.M., Kaputa K., Farma J.M., Abbas A.E.-S. (2017). Application of 3D Tumoroid Systems to Define Immune and Cytotoxic Therapeutic Responses Based on Tumoroid and Tissue Slice Culture Molecular Signatures. Oncotarget.

[114] Neal J.T., Li X., Zhu J., Giangarra V., Grzeskowiak C.L., Ju J., Liu I.H., Chiou S.-H., Salahudeen A.A., Smith A.R. (2018). Organoid Modeling of the Tumor Immune Microenvironment. Cell.

[115] You D., Hillerman S., Locke G., Chaudhry C., Stromko C., Murtaza A., Fan Y., Koenitzer J., Chen Y., Briceno S. (2021). Enhanced Antitumor Immunity by a Novel Small Molecule HPK1 Inhibitor. J. Immunother. Cancer.

[116] Selby M.J., Engelhardt J.J., Johnston R.J., Lu L.-S., Han M., Thudium K., Yao D., Quigley M., Valle J., Wang C. (2016). Preclinical Development of Ipilimumab and Nivolumab Combination Immunotherapy: Mouse Tumor Models, In Vitro Functional Studies, and Cynomolgus Macaque Toxicology. PLoS ONE.

[117] Goggi J.L., Tan Y.X., Hartimath S.V., Jieu B., Hwang Y.Y., Jiang L., Boominathan R., Cheng P., Yuen T.Y., Chin H.X. (2020). Granzyme B PET Imaging of Immune Checkpoint Inhibitor Combinations in Colon Cancer Phenotypes. Mol. Imaging Biol..

[118] Um W., Ko H., You D.G., Lim S., Kwak G., Shim M.K., Yang S., Lee J., Song Y., Kim K. (2020). Necroptosis-Inducible Polymeric Nanobubbles for Enhanced Cancer Sonoimmunotherapy. Adv. Mater..

[119] Kristensen L.K., Fröhlich C., Christensen C., Melander M.C., Poulsen T.T., Galler G.R., Lantto J., Horak I.D., Kragh M., Nielsen C.H. (2019). CD4+ and CD8a+ PET Imaging Predicts Response to Novel PD-1 Checkpoint Inhibitor: Studies of Sym021 in Syngeneic Mouse Cancer Models. Theranostics.

[120] Napolitano S., Matrone N., Muddassir A.L., Martini G., Sorokin A., De Falco V., Giunta E.F., Ciardiello D., Martinelli E., Belli V. (2019). Triple Blockade of EGFR, MEK and PD-L1 Has Antitumor Activity in Colorectal Cancer Models with Constitutive Activation of MAPK Signaling and PD-L1 Overexpression. J. Exp. Clin. Cancer Res..

[121] Schweickert P.G., Yang Y., White E.E., Cresswell G.M., Elzey B.D., Ratliff T.L., Arumugam P., Antoniak S., Mackman N., Flick M.J. (2021). Thrombin-PAR1 Signaling in Pancreatic Cancer Promotes an Immunosuppressive Microenvironment. J. Thromb. Haemost..

[122] Kim J.S., Kim E.J., Lee S., Tan X., Liu X., Park S., Kang K., Yoon J.-S., Ko Y.H., Kurie J.M. (2019). MiR-34a and MiR-34b/c Have Distinct Effects on the Suppression of Lung Adenocarcinomas. Exp. Mol. Med..

[123] Juneja V.R., McGuire K.A., Manguso R.T., LaFleur M.W., Collins N., Haining W.N., Freeman G.J., Sharpe A.H. (2017). PD-L1 on Tumor Cells Is Sufficient for Immune Evasion in Immunogenic Tumors and Inhibits CD8 T Cell Cytotoxicity. J. Exp. Med..

[124] Vandeveer A.J., Fallon J.K., Tighe R., Sabzevari H., Schlom J., Greiner J.W. (2016). Systemic Immunotherapy of Non-Muscle Invasive Mouse Bladder Cancer with Avelumab, an Anti-PD-L1 Immune Checkpoint Inhibitor. Cancer Immunol. Res..

[125] Rigo V., Emionite L., Daga A., Astigiano S., Corrias M.V., Quintarelli C., Locatelli F., Ferrini S., Croce M. (2017). Combined Immunotherapy with Anti-PDL-1/PD-1 and Anti-CD4 Antibodies Cures Syngeneic Disseminated Neuroblastoma. Sci. Rep..

[126] Yakkundi P., Gonsalves E., Galou-Lameyer M., Selby M.J., Chan W.K. (2019). Aryl Hydrocarbon Receptor Acts as a Tumor Suppressor in a Syngeneic MC38 Colon Carcinoma Tumor Model. Hypoxia.

[127] Zhong W., Myers J.S., Wang F., Wang K., Lucas J., Rosfjord E., Lucas J., Hooper A.T., Yang S., Lemon L.A. (2020). Comparison of the Molecular and Cellular Phenotypes of Common Mouse Syngeneic Models with Human Tumors. BMC Genom..

[128] Castle J.C., Loewer M., Boegel S., de Graaf J., Bender C., Tadmor A.D., Boisguerin V., Bukur T., Sorn P., Paret C. (2014). Immunomic, Genomic and Transcriptomic Characterization of CT26 Colorectal Carcinoma. BMC Genom..

[129] Gentles A.J., Newman A.M., Liu C.L., Bratman S.V., Feng W., Kim D., Nair V.S., Xu Y., Khuong A., Hoang C.D. (2015). The Prognostic Landscape of Genes and Infiltrating Immune Cells across Human Cancers. Nat. Med..

[130] Mosely S.I.S., Prime J.E., Sainson R.C.A., Koopmann J.-O., Wang D.Y.Q., Greenawalt D.M., Ahdesmaki M.J., Leyland R., Mullins S., Pacelli L. (2017). Rational Selection of Syngeneic Preclinical Tumor Models for Immunotherapeutic Drug Discovery. Cancer Immunol. Res..

[131] Liu T., Zhang X., Du L., Wang Y., Liu X., Tian H., Wang L., Li P., Zhao Y., Duan W. (2019). Exosome-Transmitted MiR-128-3p Increase Chemosensitivity of Oxaliplatin-Resistant Colorectal Cancer. Mol. Cancer.

[132] Hsu H.-H., Chen M.-C., Baskaran R., Lin Y.-M., Day C.H., Lin Y.-J., Tu C.-C., Vijaya Padma V., Kuo W.-W., Huang C.-Y. (2018). Oxaliplatin Resistance in Colorectal Cancer Cells Is Mediated via Activation of ABCG2 to Alleviate ER Stress Induced Apoptosis. J. Cell. Physiol..

[133] Tesniere A., Schlemmer F., Boige V., Kepp O., Martins I., Ghiringhelli F., Aymeric L., Michaud M., Apetoh L., Barault L. (2010). Immunogenic Death of Colon Cancer Cells Treated with Oxaliplatin. Oncogene.

[134] Taniura T., Iida Y., Kotani H., Ishitobi K., Tajima Y., Harada M. (2020). Immunogenic Chemotherapy in Two Mouse Colon Cancer Models. Cancer Sci..

[135] Combès E., Andrade A.F., Tosi D., Michaud H.-A., Coquel F., Garambois V., Desigaud D., Jarlier M., Coquelle A., Pasero P. (2019). Inhibition of Ataxia-Telangiectasia Mutated and RAD3-Related (ATR) Overcomes Oxaliplatin Resistance and Promotes Antitumor Immunity in Colorectal Cancer. Cancer Res..

[136] Golchin S., Alimohammadi R., Rostami Nejad M., Jalali S.A. (2019). Synergistic Antitumor Effect of Anti-PD-L1 Combined with Oxaliplatin on a Mouse Tumor Model. J. Cell. Physiol..

[137] Eng C., Kim T.W., Bendell J., Argilés G., Tebbutt N.C., Di Bartolomeo M., Falcone A., Fakih M., Kozloff M., Segal N.H. (2019). Atezolizumab with or without Cobimetinib versus Regorafenib in Previously Treated Metastatic Colorectal Cancer (IMblaze370): A Multicentre, Open-Label, Phase 3, Randomised, Controlled Trial. Lancet Oncol..

[138] Yu J., Green M.D., Li S., Sun Y., Journey S.N., Choi J.E., Rizvi S.M., Qin A., Waninger J.J., Lang X. (2021). Liver Metastasis Restrains Immunotherapy Efficacy via Macrophage-Mediated T Cell Elimination. Nat. Med..

[139] Pietrocola F., Pol J., Vacchelli E., Rao S., Enot D.P., Baracco E.E., Levesque S., Castoldi F., Jacquelot N., Yamazaki T. (2016). Caloric Restriction Mimetics Enhance Anticancer Immunosurveillance. Cancer Cell.

[140] Liu X., Pu Y., Cron K., Deng L., Kline J., Frazier W.A., Xu H., Peng H., Fu Y.-X., Xu M.M. (2015). CD47 Blockade Triggers T Cell-Mediated Destruction of Immunogenic Tumors. Nat. Med..

[141] Lu W., Yu W., He J., Liu W., Yang J., Lin X., Zhang Y., Wang X., Jiang W., Luo J. (2020). Reprogramming Immunosuppressive Myeloid Cells Facilitates Immunotherapy for Colorectal Cancer. EMBO Mol. Med..

[142] Mestas J., Hughes C.C.W. (2004). Of Mice and Not Men: Differences between Mouse and Human Immunology. J. Immunol..

[143] Shultz L.D., Brehm M.A., Garcia-Martinez J.V., Greiner D.L. (2012). Humanized Mice for Immune System Investigation: Progress, Promise and Challenges. Nat. Rev. Immunol..

[144] Huntington N.D., Di Santo J.P. (2008). Humanized Immune System (HIS) Mice as a Tool to Study Human NK Cell Development. Curr. Top. Microbiol. Immunol..

[145] Wege A.K. (2018). Humanized Mouse Models for the Preclinical Assessment of Cancer Immunotherapy. BioDrugs Clin. Immunother. Biopharm. Gene Ther..

[146] Okada S., Vaeteewoottacharn K., Kariya R. (2018). Establishment of a Patient-Derived Tumor Xenograft Model and Application for Precision Cancer Medicine. Chem. Pharm. Bull..

[147] Walsh N.C., Kenney L.L., Jangalwe S., Aryee K.-E., Greiner D.L., Brehm M.A., Shultz L.D. (2017). Humanized Mouse Models of Clinical Disease. Annu. Rev. Pathol..

[148] Ali N., Flutter B., Sanchez Rodriguez R., Sharif-Paghaleh E., Barber L.D., Lombardi G., Nestle F.O. (2012). Xenogeneic Graft-versus-Host-Disease in NOD-Scid IL-2Rγnull Mice Display a T-Effector Memory Phenotype. PLoS ONE.

[149] Ito R., Takahashi T., Ito M. (2018). Humanized Mouse Models: Application to Human Diseases. J. Cell. Physiol..

[150] De La Rochere P., Guil-Luna S., Decaudin D., Azar G., Sidhu S.S., Piaggio E. (2018). Humanized Mice for the Study of Immuno-Oncology. Trends Immunol..

[151] Allen T.M., Brehm M.A., Bridges S., Ferguson S., Kumar P., Mirochnitchenko O., Palucka K., Pelanda R., Sanders-Beer B., Shultz L.D. (2019). Humanized Immune System Mouse Models: Progress, Challenges and Opportunities. Nat. Immunol..

[152] Wang M., Yao L.-C., Cheng M., Cai D., Martinek J., Pan C.-X., Shi W., Ma A.-H., De Vere White R.W., Airhart S. (2018). Humanized Mice in Studying Efficacy and Mechanisms of PD-1-Targeted Cancer Immunotherapy. FASEB J..

[153] Klichinsky M., Ruella M., Shestova O., Lu X.M., Best A., Zeeman M., Schmierer M., Gabrusiewicz K., Anderson N.R., Petty N.E. (2020). Human Chimeric Antigen Receptor Macrophages for Cancer Immunotherapy. Nat. Biotechnol..

[154] Yao L.-C., Aryee K.-E., Cheng M., Kaur P., Keck J.G., Brehm M.A. (2019). Creation of PDX-Bearing Humanized Mice to Study Immuno-Oncology. Methods Mol. Biol..

[155] Lazzari L., Corti G., Picco G., Isella C., Montone M., Arcella P., Durinikova E., Zanella E.R., Novara L., Barbosa F. (2019). Patient-Derived Xenografts and Matched Cell Lines Identify Pharmacogenomic Vulnerabilities in Colorectal Cancer. Clin. Cancer Res..

[156] Inoue A., Deem A.K., Kopetz S., Heffernan T.P., Draetta G.F., Carugo A. (2019). Current and Future Horizons of Patient-Derived Xenograft Models in Colorectal Cancer Translational Research. Cancers.

[157] Zhang Y., Lee S.H., Wang C., Gao Y., Li J., Xu W. (2020). Establishing Metastatic Patient-Derived Xenograft Model for Colorectal Cancer. Jpn. J. Clin. Oncol..

[158] Hidalgo M., Amant F., Biankin A.V., Budinská E., Byrne A.T., Caldas C., Clarke R.B., de Jong S., Jonkers J., Mælandsmo G.M. (2014). Patient-Derived Xenograft Models: An Emerging Platform for Translational Cancer Research. Cancer Discov..

[159] Gitto S.B., Kim H., Rafail S., Omran D.K., Medvedev S., Kinose Y., Rodriguez-Garcia A., Flowers A.J., Xu H., Schwartz L.E. (2020). An Autologous Humanized Patient-Derived-Xenograft Platform to Evaluate Immunotherapy in Ovarian Cancer. Gynecol. Oncol..

[160] Capasso A., Lang J., Pitts T.M., Jordan K.R., Lieu C.H., Davis S.L., Diamond J.R., Kopetz S., Barbee J., Peterson J. (2019). Characterization of Immune Responses to Anti-PD-1 Mono and Combination Immunotherapy in Hematopoietic Humanized Mice Implanted with Tumor Xenografts. J. Immunother. Cancer.

[161] Chan T., Wiltrout R.H., Weiss J.M. (2011). Immunotherapeutic Modulation of the Suppressive Liver and Tumor Microenvironments. Int. Immunopharmacol..

[162] Halama N., Spille A., Lerchl T., Brand K., Herpel E., Welte S., Keim S., Lahrmann B., Klupp F., Kahlert C. (2013). Hepatic Metastases of Colorectal Cancer Are Rather Homogeneous but Differ from Primary Lesions in Terms of Immune Cell Infiltration. Oncoimmunology.

[163] Skardal A., Shupe T., Atala A. (2016). Organoid-on-a-Chip and Body-on-a-Chip Systems for Drug Screening and Disease Modeling. Drug Discov. Today.

[164] Duzagac F., Saorin G., Memeo L., Canzonieri V., Rizzolio F. (2021). Microfluidic Organoids-on-a-Chip: Quantum Leap in Cancer Research. Cancers.

[165] Skardal A., Devarasetty M., Forsythe S., Atala A., Soker S. (2016). A Reductionist Metastasis-on-a-Chip Platform for in Vitro Tumor Progression Modeling and Drug Screening. Biotechnol. Bioeng..

[166] Parlato S., De Ninno A., Molfetta R., Toschi E., Salerno D., Mencattini A., Romagnoli G., Fragale A., Roccazzello L., Buoncervello M. (2017). 3D Microfluidic Model for Evaluating Immunotherapy Efficacy by Tracking Dendritic Cell Behaviour toward Tumor Cells. Sci. Rep..

[167] Miller C.P., Shin W., Ahn E.H., Kim H.J., Kim D.-H. (2020). Engineering Microphysiological Immune System Responses on Chips. Trends Biotechnol..

